# A fast and efficient python library for interfacing with the Biological Magnetic Resonance Data Bank

**DOI:** 10.1186/s12859-017-1580-5

**Published:** 2017-03-17

**Authors:** Andrey Smelter, Morgan Astra, Hunter N. B. Moseley

**Affiliations:** 1 School of Interdisciplinary and Graduate Studies, University of Louisville, Louisville, KY 40292 USA; 2 Department of Computer Engineering and Computer Science, University of Louisville, Louisville, KY 40292 USA; 3 Department of Molecular and Cellular Biochemistry, University of Kentucky, Lexington, KY 40356 USA; 4 Markey Cancer Center, University of Kentucky, Lexington, KY 40356 USA; 5 Center for Environmental and Systems Biochemistry, University of Kentucky, Lexington, KY 40356 USA; 6 Institute for Biomedical Informatics, University of Kentucky, Lexington, KY 40356 USA

**Keywords:** Biological Magnetic Resonance Bank, Nuclear magnetic resonance, NMR-STAR, JSON, nmrstarlib, Python

## Abstract

**Background:**

The Biological Magnetic Resonance Data Bank (BMRB) is a public repository of Nuclear Magnetic Resonance (NMR) spectroscopic data of biological macromolecules. It is an important resource for many researchers using NMR to study structural, biophysical, and biochemical properties of biological macromolecules. It is primarily maintained and accessed in a flat file ASCII format known as NMR-STAR. While the format is human readable, the size of most BMRB entries makes computer readability and explicit representation a practical requirement for almost any rigorous systematic analysis.

**Results:**

To aid in the use of this public resource, we have developed a package called nmrstarlib in the popular open-source programming language Python. The nmrstarlib’s implementation is very efficient, both in design and execution. The library has facilities for reading and writing both NMR-STAR version 2.1 and 3.1 formatted files, parsing them into usable Python dictionary- and list-based data structures, making access and manipulation of the experimental data very natural within Python programs (i.e. “saveframe” and “loop” records represented as individual Python dictionary data structures). Another major advantage of this design is that data stored in original NMR-STAR can be easily converted into its equivalent JavaScript Object Notation (JSON) format, a lightweight data interchange format, facilitating data access and manipulation using Python and any other programming language that implements a JSON parser/generator (i.e., all popular programming languages). We have also developed tools to visualize assigned chemical shift values and to convert between NMR-STAR and JSONized NMR-STAR formatted files. Full API Reference Documentation, User Guide and Tutorial with code examples are also available.

We have tested this new library on all current BMRB entries: 100% of all entries are parsed without any errors for both NMR-STAR version 2.1 and version 3.1 formatted files. We also compared our software to three currently available Python libraries for parsing NMR-STAR formatted files: PyStarLib, NMRPyStar, and PyNMRSTAR.

**Conclusions:**

The nmrstarlib package is a simple, fast, and efficient library for accessing data from the BMRB. The library provides an intuitive dictionary-based interface with which Python programs can read, edit, and write NMR-STAR formatted files and their equivalent JSONized NMR-STAR files. The nmrstarlib package can be used as a library for accessing and manipulating data stored in NMR-STAR files and as a command-line tool to convert from NMR-STAR file format into its equivalent JSON file format and vice versa, and to visualize chemical shift values. Furthermore, the nmrstarlib implementation provides a guide for effectively JSONizing other older scientific formats, improving the FAIRness of data in these formats.

**Electronic supplementary material:**

The online version of this article (doi:10.1186/s12859-017-1580-5) contains supplementary material, which is available to authorized users.

## Background

The Biological Magnetic Resonance Data Bank (BMRB) is a free, publicly-accessible repository of data on peptides, proteins, and nucleic acids obtained through NMR Spectroscopy [1], that is part of the worldwide Protein Databank (wwPDB) [2]. It currently consists of more than 11,000 individual NMR-STAR file entries, containing a wide range of NMR spectral data, experimental details, and biochemical data collected from thousands of biological samples. The NMR-STAR format is based on the Self-defining Text Archival and Retrieving (STAR) flat file database format [3], with some modifications specific to the BMRB. STAR provides a hierarchical dictionary structure for storing arbitrary data. In NMR-STAR, the format specifies top-level dictionaries called “saveframes”, which are used to categorize the data and meta-data about the experiment. Inside each saveframe is an arbitrarily number of key-value pairs and tables of records (loops). The key-value pairs store a single piece of information under a descriptive variable name. Each loop stores a table of records, each record containing a set of values representing individual fields in the record. There are currently two active versions of the BMRB: version 2.1 and version 3.1. While they both use the same NMR-STAR format at the most general level, the layout of the data in the two formats is different.

Python is a free, open-source scripting language which runs on all major operating systems [4, 5]. It is designed to facilitate the development and maintenance of simple, efficient, and readable code. Python has object-oriented programming facilities and includes several high-level data structure objects in its standard library. Among these are the dictionary, a data structure implemented via the dict class that stores data as a set of key-value pairs (specific mappings between keys and values). The OrderedDict class is identical to the dict class except that the order of inserted keys-value pairs is remembered. This is particularly useful for categorical data with sequential relationships. The dictionary data structure is the most straightforward mechanism for representing and using data from NMR-STAR files, which have a nested, mostly dictionary-like structure themselves. However, to our knowledge no NMR-STAR parsing library using this design exists. The newest major version of Python (version 3.0.0), was initially released on 2008-12-03, however many software libraries and utilities written in Python still use Python version 2.x exclusively. As Python version 3.1 brings many substantial improvements over Python 2.x (including the addition of the OrderedDict class, which was later back-ported to Python version 2.7 [6]). As of Python version 3.5 OrderedDict is implemented in C which makes it much faster than the Python 2.7 implementation of OrderedDict. Moreover in Python 3.6, the dict data structure implementation becomes ordered by default and dict and OrderedDict are more efficient than in any previous versions of Python. While we provide support for Python 2.7 for use by legacy code, we believe that researchers will prefer libraries and tools written in latest version of Python in order to develop maintainable codebases, especially as Python version 2.x becomes less supported over time. Moreover, Python version 2.7 will no longer be maintained after Spring of 2020 [7]. Two publically available Python libraries for parsing NMR-STAR format files PyStarLib [8] and NMRPyStar [9] both require Python version 2.7. PyNMRSTAR [10] works with both major versions of Python (2.7 and 3.3+).

## Implementation

**Fig. 1:**
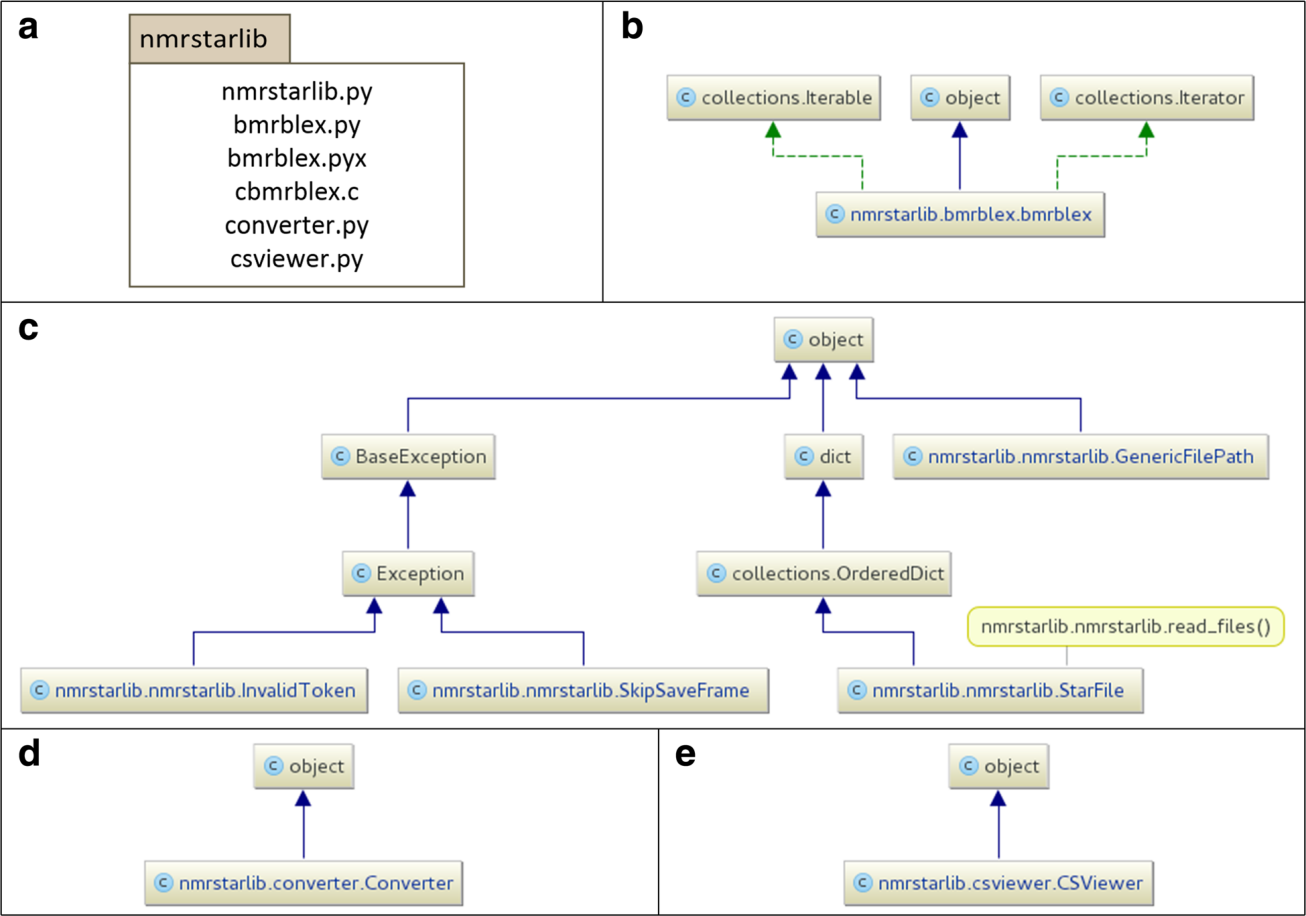
Organization of the nmrstarlib package version 1.1.0. **a** UML package diagram of the nmrstarlib library; **b** UML class diagram of the bmrblex.py (bmrblex.pyx) module; **c** UML class diagram of the nmrstarlib.py module; **d** UML class diagram of the converter.py module; **e** UML class diagram of the csviewer.py module

The nmrstarlib package consists of several modules: nmrstarlib.py, bmrblex.py, converter.py, and csviewer.py (Fig. 1a). The nmrstarlib module (Fig. 1c) provides the StarFile class, which implements a nested Python dictionary/list representation of a BMRB NMR-STAR file. Once a NMR-STAR formatted file is processed into a StarFile object, experimental data can be accessed directly from the StarFile object, using bracket accessors as with any regular Python dict object. The nmrstarlib module relies on the bmrblex module (Fig. 1b) for processing of tokens. The bmrblex module provides the bmrblex generator – BMRB lexical analyzer (parser). We provide two versions of the bmrblex module: a pure Python version (bmrblex.py) and a Python + C extension (bmrblex.py, cbmrblex.c) for faster performance. The compiled C extensions are implemented in the Cython programming language [11], which we will call the Cython implementaion. If the Cython implementation of bmrblex fails for any reason, the library will use the Python implementation, ensuring that the library always works.

The library creates an internal representation of the NMR-STAR format as a nesting of OrderedDict objects with the top-level object StarFile inheriting from the OrderedDict class (Fig. 1c). This allows the user to access data in its original NMR-STAR organization using familiar Python dictionary syntax. The library provides facilities to read data from NMR-STAR formatted files into an internal StarFile object, to access and make modifications to this StarFile object, and to save the resulting StarFile object as a new NMR-STAR formatted file. It is also possible to create NMR-STAR files from scratch using this library; however, this requires the user to adhere to the recommended layout for NMR-STAR formatted files by adding keys and values to the StarFile object in the appropriate order.

**Fig. 2:**
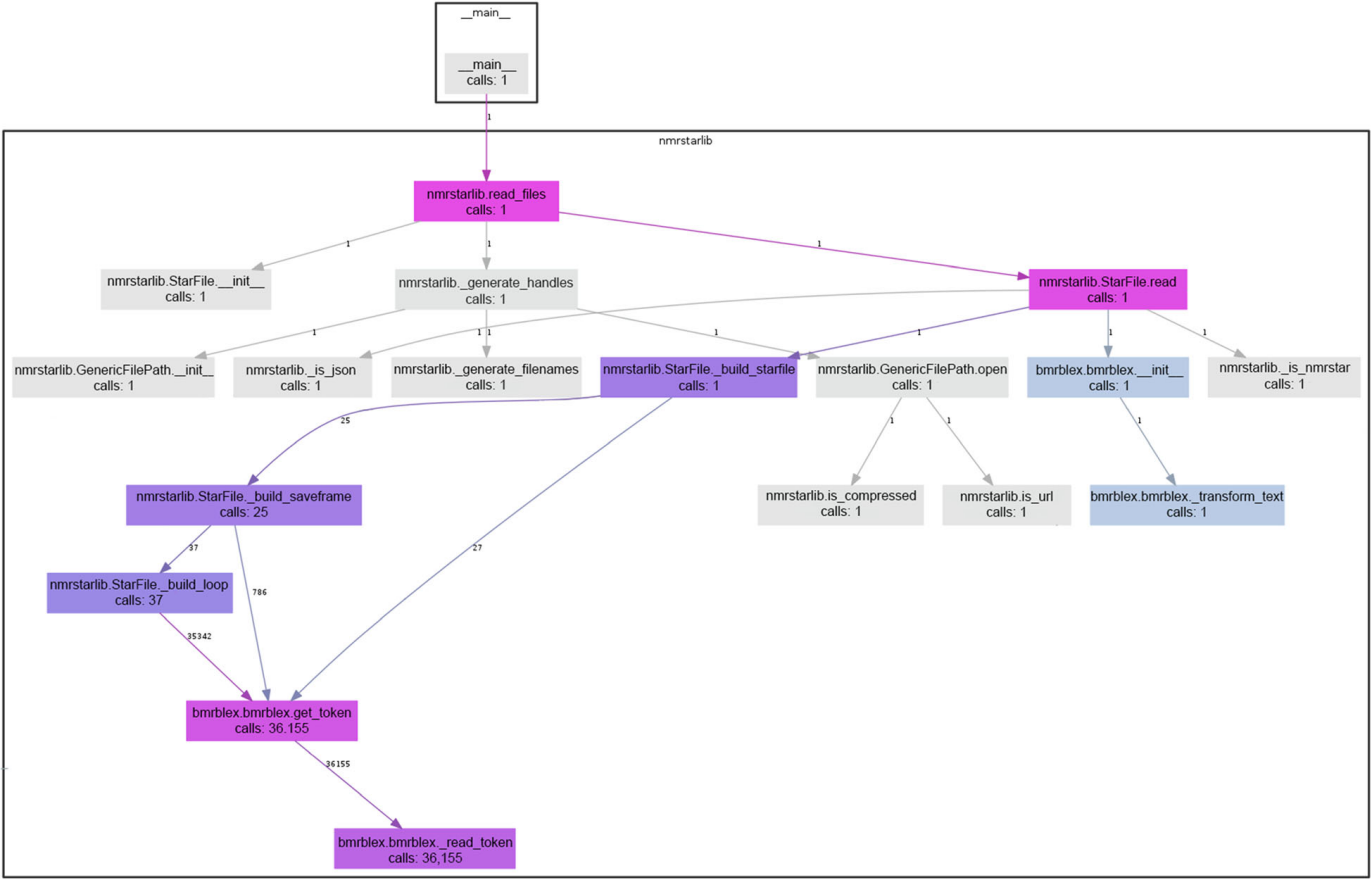
Diagram showing what function calls are made during the process of StarFile object creation

The nmrstarlib module provides a memory-efficient read_files() generator function (Fig. 1c) that yields (emits) StarFile objects, one at a time for each file parsed. When reading an NMR-STAR formatted file (Fig. 2, Additional files 1 and 2), the read_files() generator function first opens the file and passes a filehandle to the StarFile.read() method that reads the text into Python as a string and passes that string into the bmrblex object that then splits the text into tokens. As the bmrblex lexical analyzer keeps emitting valid tokens, the StarFile object is constructed sequentially. The StarFile object decides what type of token it is dealing with and chooses which internal method to call in order to construct itself, i.e. calls to StarFile._build_starfile(), Starfile._build_saveframe(), or StarFile._build_loop(). For example, Fig. 2 shows the function call diagram during the StarFile object creation: the _build_saveframe() method is called 25 times and _build_loop() is called 37 times, meaning that the NMR-STAR file consists of 25 different saveframe categories and 37 loops. The total number of tokens processed is equal to 36,155 = 27 (from _build_starfile) + 786 (from _build_saveframe) + 35,342 (from _build_loop).

**Fig. 3:**
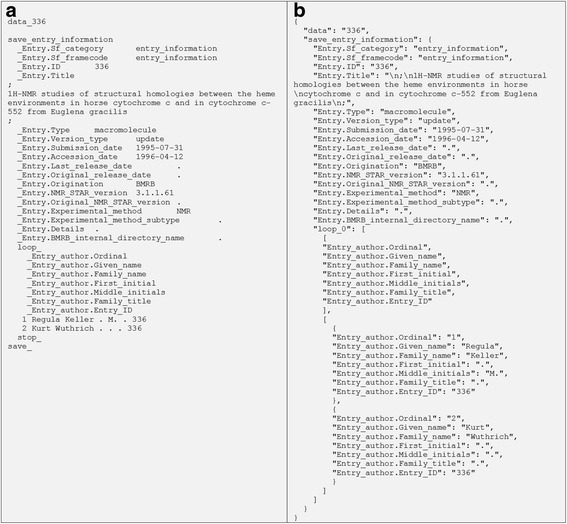
Internal StarFile object representation and correspondence to NMR-STAR format without comments: **a** An example of a NMR-STAR formatted file; **b** StarFile dictionary representation equivalent to the NMR-STAR formatted file and the JSONized version of the NMR-STAR file

Each saveframe category is also an OrderedDict data structure that can be accessed by saveframe name as the key from the top-level StarFile object. Once a saveframe dictionary is constructed and populated with key-value pairs, it descends further into each loop and constructs a tuple of two lists: the first list corresponding to loop field keys (loop field names); the second list consists of OrderedDict objects corresponding to loop rows (loop records) in the original NMR-STAR file. By the end of parsing, a single nested dictionary/list structure in the form of a StarFile dictionary object (Fig. 3b) is constructed, emulating the structure of the original NMR-STAR formatted file (Fig. 3a). In addition, comments can be parsed and included as additional key-value pairs within the nested dictionary structure.

The nmrstarlib module provides a GenericFilePath (Figs. 1c and 2) object that is used by the read_files() generator function in order to open NMR-STAR formatted files from many different sources: a single file on a local machine; a URL address of a single file; a directory of files on a local machine; an archive of files on a local machine; a URL address of an archive of files; or the BMRB id of a single file.

To write from a StarFile object to an NMR-STAR formatted file, the library recursively crawls through the StarFile dictionary structure, formatting and printing each of the keys and corresponding values sequentially. This allows nmrstarlib to recall the sequential order of the original NMR-STAR formatted file, due to the stored ordering of key insertion from the underlying OrderedDict objects. Using Python’s json library, the entire StarFile dictionary structure can be saved as JSON (JavaScript Object Notation), which is an open, human-readable, lightweight data exchange format that is readable by most programming languages via optimized parsing libraries. This JSON conversion of StarFile objects greatly facilitated the implementation of the converter module which converts original NMR-STAR formatted files into their equivalent JSONized NMR-STAR files and vice versa. The converter module (Fig. 1d) consists of a single Converter class which can convert in both one-to-one (single file) and many-to-many (directory or archive of files) modes. See “The nmrstarlib API Reference” documentation of the converter module for the full list of available conversion options (Additional file 3).

**Fig. 4:**
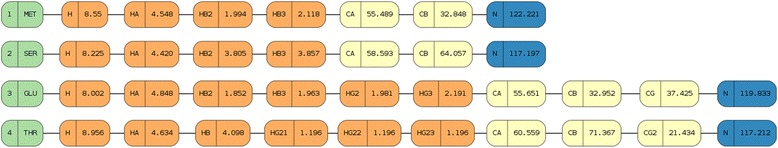
Example of output file: chemical shifts organized by amino acid residue type produced by csviewer module

In order to simplify access to assigned chemical shift data, we created the csviewer module (Fig. 1e) that includes the CSViewer class that can access both the NMR-STAR version 2.1 and version 3.1 assigned chemical shifts loop and visualize (organize) chemical shift values by amino acid residue type, and save this visualization as an image file or a pdf document (Fig. 4). The csviewer module requires the graphviz Python library [12] in order to create an output file. In addition to visualizing chemical shift values, the csviewer module provide code example for utilizing the nmrstarlib library.

Overall, the nmrstarlib package can be used in two ways: 1) as a library for accessing and manipulating data stored in NMR-STAR formatted files, converting between NMR-STAR and its equivalent JSON format, and visualizing assigned chemical shift values; or 2) as a standalone command-line tool for converting files in bulk and visualizing assigned chemical shift values. We used the docopt Python library [13] to create the nmrstarlib package command-line interface.

## Results

### Performance on NMR-STAR formatted files

**Fig. 5:**
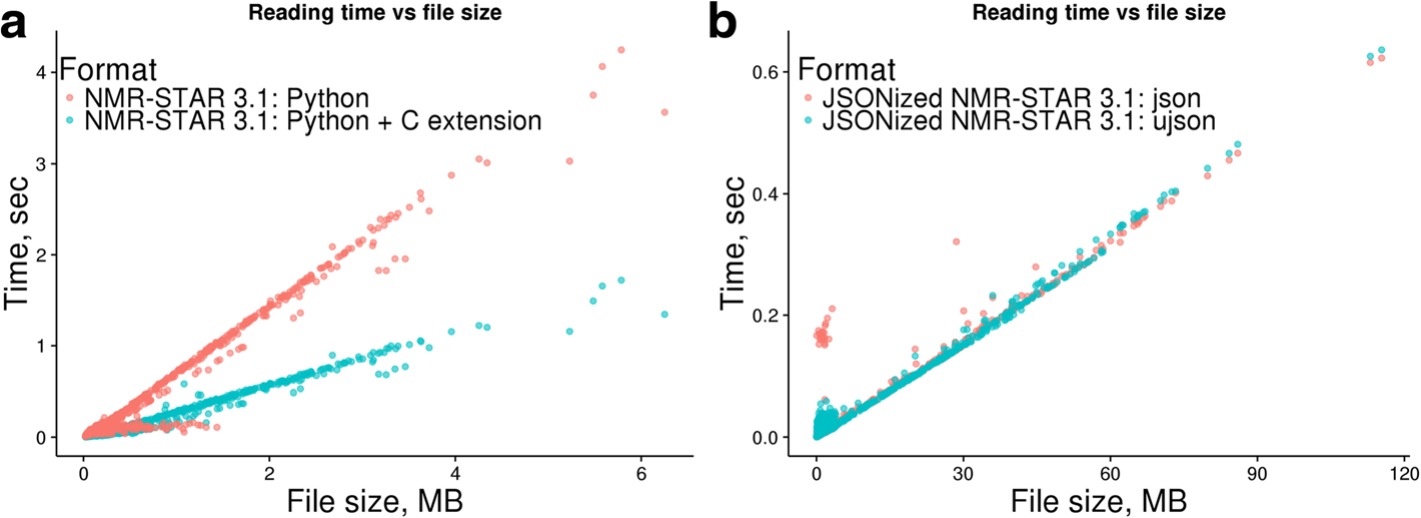
Graph showing the dependency of loading time into StarFile object from the size of file: **a** Loading times for NMR-STAR 3.1 formatted files; **b** Loading times for JSONized NMR-STAR 3.1 files

As part of nmrstarlib’s development process, we tested our library extensively against the entire BMRB (as of December 11, 2016) for both NMR-STAR version 2.1 and version 3.1 [14]. To measure the performance speed of the nmrstarlib library, we used a simple program that accesses NMR-STAR files from local directory one file at a time, which then creates a StarFile object and records how much time in seconds it took to create the object. Table 1 shows that our library was able to read the entire BMRB for both NMR-STAR version 2.1 and version 3.1 without any errors. With the pure Python implementation, it took 1,110 s (~18.3 min) and 326 s (~5.4 min) to read NMR-STAR version 3.1 and NMR-STAR version 2.1, respectively. With the more efficient Cython implementation, it took 423 s (~7 min) and 320 s (~5.3 min) to read NMR-STAR version 3.1 and NMR-STAR version 2.1, respectively. We used the metric kilobytes per second (KB/sec), because files/sec would be a misleading metric due to widely varying files sizes in the BMRB and because read times scale almost linearly (Fig. 5) with file size. As such, we found that nmrstarlib’s average reading speed is 1,700 KB/sec (NMR-STAR 3.1) and 3,290 KB/sec (NMR-STAR 2.1) for the Python implementation and 4,421 KB/sec (NMR-STAR 3.1) and 3,351 KB/sec (NMR-STAR 2.1) for the Cython implementation on the hardware used for testing. The NMR-STAR 3.1 is more comprehensive than NMR-STAR 2.1 and usually represents more experimental information and details. This additional complexity is computationally harder to parse. However, for our Cython implementation average reading speed for NMR-STAR 3.1 was faster than for NMR-STAR 2.1 due to multiline text pre-processing discussed in more detail in the next section.

**Table 1:** The nmrstarlib library performance test against NMR-STAR formatted files using pure Python and Python with C extension and against JSONized NMR-STAR files using the standard Python library json parser and the UltraJSON (ujson) 3^rd^ party library

			NMR-STAR 2.1	NMR-STAR 3.1	JSONized NMR-STAR 2.1	JSONized NMR-STAR 3.1
Number of files			11,270	11,244	11,270	11,244
Total size of files, GB			1.1	1.8	4.6	22.0
Time, sec	Pure Python	json	326	1,100	30	130
	Python with C extension	^a^ujson	320	423	27	126
Average reading speed, KB/sec	Pure Python	json	3,290	1,700	158,549	176,479
	Python with C extension	^a^ujson	3,351	4,421	176,166	182,082

^a^We added support for the ujson library for versions of Python starting with Python 3.6, because the ujson library does not provide methods to keep the dict data structure in order when parsing from JSON files; however, starting with Python 3.6, the dict data structure is ordered by default

### Performance on JSONized NMR-STAR files

**Fig. 6:**
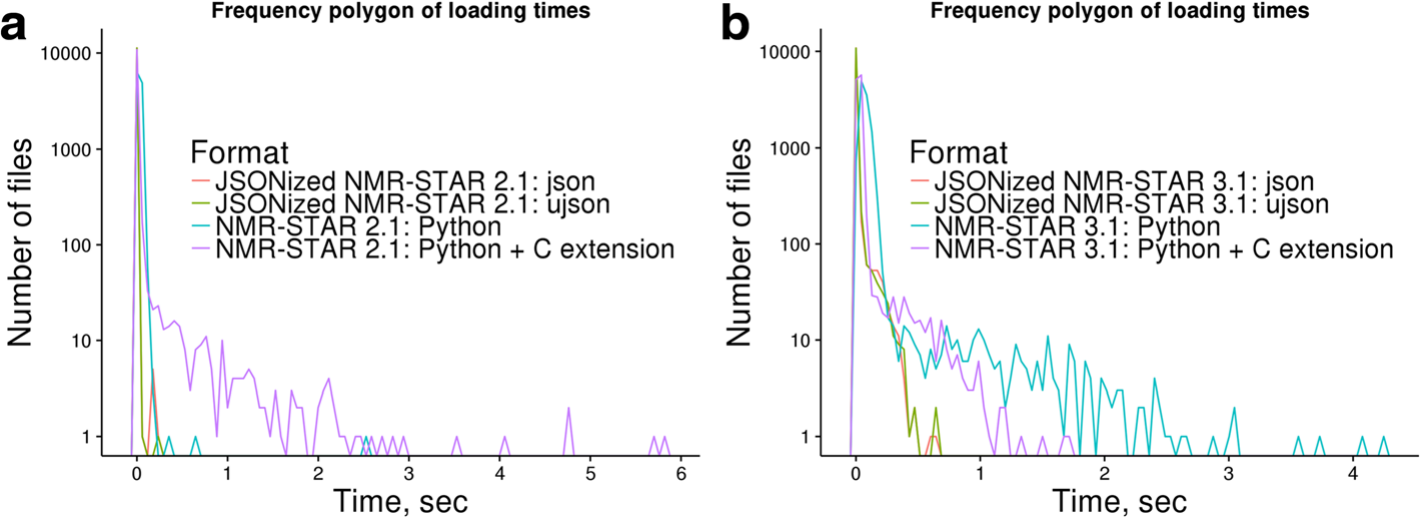
Frequency polygon of loading times for NMR-STAR files: **a** Comparison of loading times between NMR-STAR 2.1 and JSONized NMR-STAR 2.1; **b** Comparison of loading times between NMR-STAR 3.1 and JSONized NMR-STAR 3.1

Next, we converted both NMR-STAR version 2.1 and version 3.1 files into their equivalent JSON format and performed speed tests again (Table 1). We found that read times of both JSONized NMR-STAR version 2.1 and version 3.1 were significantly faster than read times of the original NMR-STAR formatted files: 130 s (~2.2 min) and 30 s (~0.5 min) for NMR-STAR version 3.1 and NMR-STAR version 2.1, respectively, for the entire BMRB data set. The average read speed was 176,479 KB/sec and 158,549 KB/sec for version 3.1 and version 2.1, respectively. Next, we tested performance using another compiled JSON parsing third-party library, UltraJSON (ujson) [15]. We found that reading times and average reading speeds of JSONized NMR-STAR files were slightly faster than using the built-in json parser: 127 s (182,082 KB/sec) and 27 s (176,166 KB/sec) for version 3.1 and version 2.1 respectively (Table 1). Table 2 shows how much time it took to convert the entire BMRB into its JSONized version and how much disk space it occupied as uncompressed directory and as compressed zip and tar archives. Compressed zip and tar formats represent the entire BMRB database in a single file and save disk space. In order to simplify access, our library provides facilities to directly read NMR-STAR files from zip and tar archives without the requirement to manually decompress and separate the archive into separate files first. Frequency polygons of loading times on Fig. 6 show that the majority of NMR-STAR and JSONized NMR-STAR files can be loaded into StarFile object in less than 1 s per file and JSONized NMR-STAR files can be loaded much faster than the original NMR-STAR files. Figure 6a and b show that the fastest reading times were for parsing JSONized NMR-STAR files using the ujson and json parsers. However on Fig. 6a, it is clear that the pure Python implementation outperformed the Cython implementation for some of the NMR-STAR 2.1 files (e.g. BMRB ID: 17192, 16692). This is because those files contain saveframe categories deposited as very large multiline blocks of text and the majority of time is spent to pre-process them, equivalent NMR-STAR 3.1 files have those saveframes properly formatted and do not require extra time to pre-process multiline text blocks. For NMR-STAR 3.1 formatted files (Fig. 6b), the Cython implementation outperformed pure Python implementation in all cases.

**Table 2:** Converting NMR-STAR formatted files into their equivalent JSON format

	Directory		zip archive		tar.gz archive		tar.bz2 archive	
Format	NMR-STAR 2.1	NMR-STAR 3.1	NMR-STAR 2.1	NMR-STAR 3.1	NMR-STAR 2.1	NMR-STAR 3.1	NMR-STAR 2.1	NMR-STAR 3.1
Number of files	11,270	11,244	11,270	11,244	11,270	11,244	11,270	11,244
Time, min	8	20	9	22	12	27	15	68
Total size, MB	4,756	22,942	230	470	200	409	131	222

### Comparison to similar existing software

Using the entire BMRB, we performed and compared speed performance tests between our nmrstarlib package and the three other publically available Python libraries for reading NMR-STAR formatted files: PyStarLib [8], NMRPyStar [9], and PyNMRSTAR [10]. For each of these libraries, we wrote a simple Python program that loads a NMR-STAR formatted file from a directory, creates an object representation, and then reports how much time it took to process each file. Results of these comparisons are summarized in Table 3. For the pure Python implementation, PyStarLib showed the fastest reading time: 239 s (~4 min) and 796 s (~13.3 min) for NMR-STAR version 2.1 and version 3.1 respectively, but it was not able to parse 0.43% (48 files) NMR-STAR version 2.1 and 4.08% (459 files) NMR-STAR version 3.1. All errors occurred inside a function that is responsible for processing multiline quoted text, which uses regular expressions to collapse multiline quoted text into a single token. The most probable cause for these errors is a regular expression that is not capable of handling all edge cases. Examples of failures include files where: i) multiline quoted text included a semicolon character inside the text; ii) multiline quoted text that is not followed by the new line character; and iii) multiline quoted text followed by a loop (see Additional files 4, 5, 6, and 7 for list of failed files as of December 11, 2016 and particular fragments of files where the failure occurred for both NMR-STAR 2.1 and NMR-STAR 3.1 formatted files).

**Table 3:** Performance comparison of nmrstarlib to other Python libraries

		nmrstarlib	PyStarLib	NMRPyStar	PyNMRSTAR
Parsing NMR-STAR 2.1					
Number of files		11,270	11,270	11,270	11,270
Time, sec	Pure Python	326	239	N/A	547
	Python with C Extension	320	N/A	N/A	144
Success rate, %		100	99.57	0	100
Parsing NMR-STAR 3.1					
Number of files		11,244	11,244	11,244	11,244
Time, sec	Pure Python	1,100	796	56,569	2,354
	Python with C Extension	423	N/A	N/A	538
Success rate, %		100	95.92	100	100

The pure Python implementation of the nmrstarlib package was the second fastest method 326 s (~5.4 min) and 1,110 s (~18.3 min) and, more importantly, parsed 100% of files for both NMR-STAR 2.1 and NMR-STAR 3.1, respectively. The NMRPyStar library showed the slowest results, taking 56,569 s (~15.7 h) to process NMR-STAR version 3.1 and was not able to read any of the NMR-STAR version 2.1 files (error status code was reported by the program during execution). Both the nmrstarlib and PyNMRSTAR provide Python + C extension implementations in order to speed up the tokenization process. The nmrstarlib performed faster than PyNMRSTAR on NMR-STAR 3.1 files: 423 s (~7 min) versus 538 s (~9 min). However, PyNMRSTAR was faster than nmrstarlib on NMR-STAR 2.1 files: 144 s (~2.4 min) versus 320 s (~5.3 min). Overall, the nmrstarlib (Python + C extension implementation) was the fastest method to read NMR-STAR 3.1 files, and PyNMRSTAR (Python + C extension implementation) was the fastest method to read NMR-STAR 2.1 files. However, when using the JSONized versions of NMR-STAR files with the nmrstarlib library, parsing speed can be further improved to 30 s for NMR-STAR 2.1 and 130 s for NMR-STAR 3.1 (see Table 1).

All tests were performed on a single workstation desktop computer with Intel(R) Core(TM) i7-4930 K CPU @ 3.40GHz processor, 64 GB memory, and a solid-state drive. The latest stable version of Python (Python 3.6.0) was used to compare libraries. Python version 2.7 was used for libraries that do not support the latest version of Python.

## Discussion

### The nmrstarlib interface

To use nmrstarlib as a library, first import the library. Next, create a StarFile generator that will return StarFile instances one at a time from many different file sources: a local file, URL address of a file, directory, archive, BMRB id. Next, the StarFile object can be utilized like any built-in Python dict object. Table 4 shows common usage patterns for reading NMR-STAR files into StarFile objects, accessing and manipulating data using bracket accessors, and writing StarFile objects back to both NMR-STAR and JSONized NMR-STAR formats. For more detailed examples, see “The nmrstarlib Tutorial” documentation (Additional file 3).

**Table 4:** Common usage patterns for the nmrstarlib module

Usage	Example
Reading:	sf_gen = nmrstarlib.read_files(‘path’) starfile = next(sf_gen)
Access/Modification:	starfile[‘saveframe’][‘key’] starfile[‘saveframe’][‘key’] = new_value
Writing:	starfile.write(fileobj, fileformat=‘nmrstar’) starfile.write(fileobj, fileformat=‘json’)

The nmrstarlib command-line interface provides two commands: convert in order to convert between NMR-STAR format and its equivalent JSON format; the csview command for quick access to assigned chemical shift data of a single StarFile, organizing chemical shifts by amino acid residue type. Table 5 shows common usage examples for the convert and csview commands. For a full list of available conversion options and more detailed examples see “The nmrstarlib API Reference” and “The nmrstarlib Tutorial” documentation. Figure 4 shows example output of the csview command.

**Table 5:** The nmrstarlib library command-line interface

Command	Description	Example
convert	Convert between NMR-STAR and JSON formats	$ python3 -m nmrstarlib convert bmr18569.str 18569.json \ --from_format=nmrstar --to_format=json $ python3 -m nmrstarlib convert 18569.json bmr18569.str \ --from_format=json --to_format=nmrstar
csview	View assigned chemical shifts	$ python3 -m nmrstarlib csview 18569 \ --csview_outfile=18569_cs_all --csview_format=png $ python3 -m nmrstarlib csview 18569 \ --aminoacids=GLU,THR --atoms=CA,CB,CG,CG2 \ --csview_outfile=18569_cs_GLU_THR_CA_CB_CG_CG2 \ --csview_format=png

We also have developed the “User Guide”, “The nmrstarlib Tutorial” and “The nmrstarlib API Reference” documentation that is available as a PDF file (Additional file 3) and up-to-date online documentation (Table 6).

**Table 6:** Comparison of nmrstarlib to other Python libraries

Feature	nmrstarlib	PyStarLib	NMRPyStar	PyNMRSTAR
Read NMR-STAR 2.1	Yes	Yes	No	Yes
Read NMR-STAR 3.1	Yes	Yes	Yes	Yes
Supported Python version	2.7, 3.4+	2.7	2.7	2.6, 2.7, 3.3+
API Reference documentation	Yes	No	No	Yes
Tutorial documentation	Yes	No	No	Yes
PDF of documentation	Yes	No	No	Yes
User Guide documentation	Yes	No	Yes	No
Up to date online documentation	Yes	No	No	No
Open Source	Yes (GitHub)	Yes (SourceForge)	Yes (GitHub)	Yes (GitHub)

### Advantages of using nmrstarlib and JSONized NMR-STAR version

One of the main advantages of our library is that it provides a one-to-one mapping between each of the following representations of BMRB entries: NMR-STAR format, internal Python OrderedDict- and list-based objects, and JSONized NMR-STAR format. This makes the library more Python-idiomatic, providing a very intuitive programming interface for accessing and manipulating NMR data. Another benefit of our nmrstarlib package is that the bmrblex lexical analyser module is written in a generic fashion, making it easy to adapt for parsing data from other STAR-related formats, for example, the Crystallographic Information File (CIF) and its closely related macromolecular CIF (mmCIF) format.

**Fig. 7:**
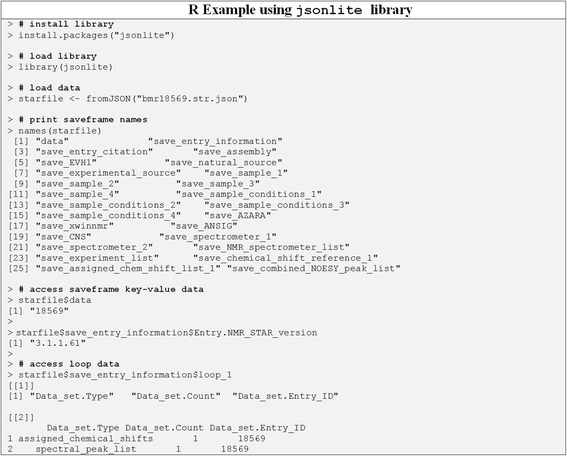
Code example showing how to access data from JSONized NMR-STAR files using R programming language

**Fig. 8:**
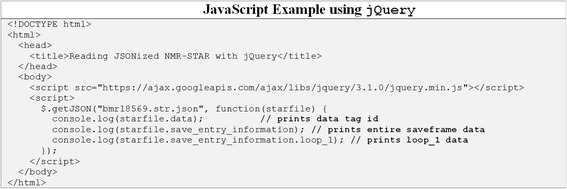
Code example showing how to access data from JSONized NMR-STAR files using JavaScript programming language

**Fig. 9:**
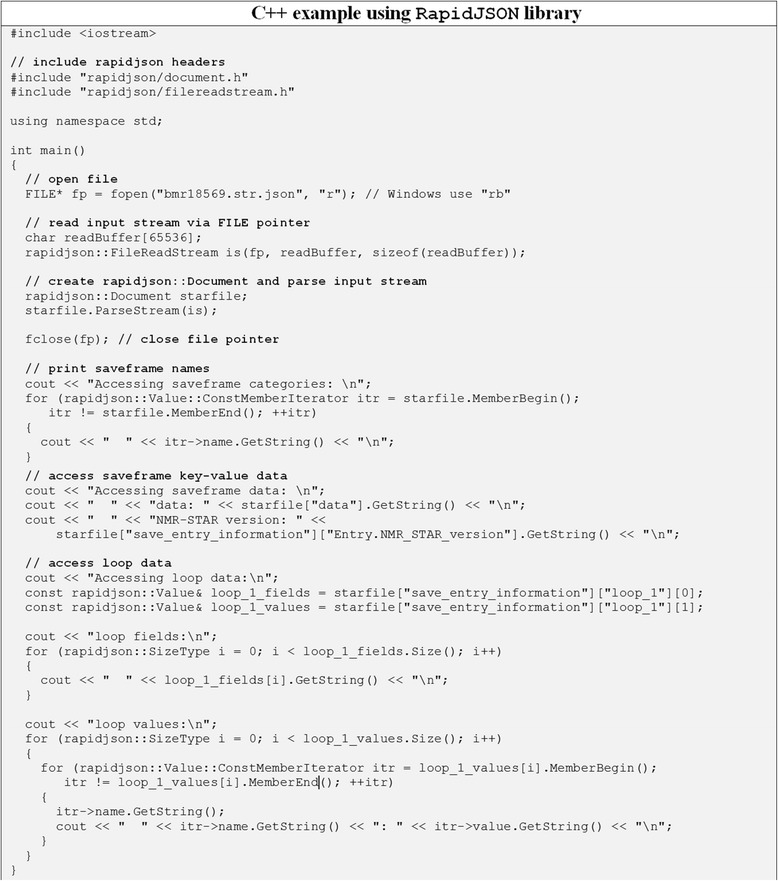
Code example showing how to access data from JSONized NMR-STAR files using C++ programming language

JSON is an open, programming language independent, human-readable, data exchange standard that represents data objects in a nested dictionary/list ASCII format. JSON is one of the most common formats for asynchronous browser/server communication as an alternative to XML (Extensible Markup Language). We selected the JSON object representation, because it has a smaller overhead compared to common XML object representations, making it faster to parse and more human-readable when formatted for this purpose. But more importantly, it facilitates a one-to-one mapping with both nested Python data structures and BMRB’s nested data representations of their entries. While XML is more flexible, it is not easily represented by a nesting of standard Python data structures that would produce an intuitive programming interface. Also, JSONization of the original NMR-STAR files provides several advantages: i) much faster reading times (see Table 1) and ii) makes the data stored in BMRB entries easily accessible to other programming languages that have JSON parsers, i.e. all modern programming languages, scripting as well as compiled, without requiring to write a specific parser for the specialized NMR-STAR format. Figures 7, 8, and 9 show code examples for accessing data from JSONized NMR-STAR files using R with the jsonlite library [16], JavaScript with the jQuery library [17], and C++ with the RapidJSON library [18] (Additional file 8 provides output of C++ example after compilation and execution), respectively.

But one disadvantage of using JSON format is that it is more verbose in comparison to the original NMR-STAR format. As a result, uncompressed JSONized NMR-STAR files occupy more disk space (Table 2). However, the nmrstarlib library offers the ability to read NMR-STAR files in both uncompressed (directory of files) and compressed (zip and tar archives) forms, making storage and access of JSONized NMR-STAR files very efficient.

## Conclusions

The nmrstarlib package is a useful Python library, providing classes and other facilities for parsing, accessing, and manipulating data stored in NMR-STAR and JSONized NMR-STAR formats. Also, nmrstarlib provides a simple command-line interface that can convert from the NMR-STAR file format into its equivalent JSON file format and vice versa, as well as accessing and visualizing assigned chemical shift values. The library has an easy-to-use, idiomatic dictionary-based interface, usable in programs written in Python. The library also has extensive documentation including the “User Guide”, “The nmrstarlib Tutorial”, and “The nmrstarlib API Reference”. Furthermore, the easy conversion into the JSONized NMR-STAR format facilitates utilization of BMRB entries by programs in any programming language with a JSON parser. This same basic approach can be used to quickly JSONize other older text-based scientific data formats, making the underlying scientific data easily accessible in a wide variety of programming languages. As demonstrated in this study, many available JSON parsers are highly optimized and typically much more efficient than specialized parsers for scientific data formats. Thus, JSONization of older scientific data formats provides easy steps for reaching Interoperability and Reusability goals of FAIR guiding principles [19].

## Additional files

Additional file 1: Function call diagram of nmrstarlib. (PNG 1167 kb)
Additional file 2: Profile of nmrstarlib execution. (TXT 27 kb)
Additional file 3: Documentation for nmrstarlib. (PDF 256 kb)
Additional file 4: List of failed NMR-STAR 2.1 files for PyStarLib. (JSON 917 bytes)
Additional file 5: List of failed NMR-STAR 3.1 files for PyStarLib. (JSON 8 kb)
Additional file 6: Fragments of failed NMR-STAR 2.1 files for PyStarLib. (TXT 16 kb)
Additional file 7: Fragments of failed NMR-STAR 3.1 files for PyStarLib. (TXT 149 kb)
Additional file 8: Output of C++ example from Fig. 9. (TXT 1 kb)

## Abbreviations

NMR: Nuclear magnetic resonance
BMRB: Biological Magnetic Resonance Data Bank
STAR: Self-defining text archival and retrieving
JSON: JavaScript Object Notation
XML: Extensible markup language
UML: Unified modeling language
