# Supplementary figures and images for: A fast and efficient python library for interfacing with the Biological Magnetic Resonance Data Bank
Source: BMC Bioinformatics. 2017 Mar 17;18:175. doi: 10.1186/s12859-017-1580-5 (PMC5356280; doi:10.1186/s12859-017-1580-5)

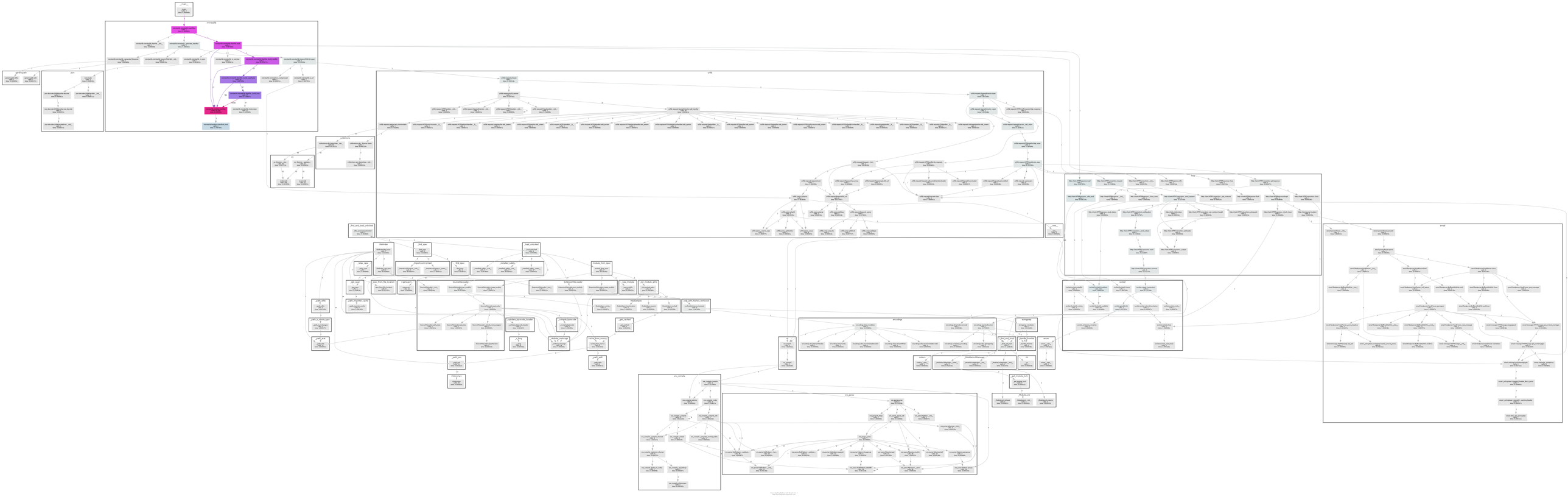

Supplement: Additional file 1: — Function call diagram of nmrstarlib. (PNG 1167 kb) [file 12859_2017_1580_MOESM1_ESM.png]
