# Supplementary material for: A fast and efficient python library for interfacing with the Biological Magnetic Resonance Data Bank
Source: BMC Bioinformatics. 2017 Mar 17;18:175. doi: 10.1186/s12859-017-1580-5 (PMC5356280; doi:10.1186/s12859-017-1580-5)
Supplement: Additional file 3: — Documentation for nmrstarlib. (PDF 256 kb) [file 12859_2017_1580_MOESM3_ESM.pdf]

nmrstarlib Documentation

Release 1.1.0

Andrey Smelter, Hunter N.B. Moseley

January 30, 2017

CONTENTS

1 nmrstarlib **1**
- 1.1 Links . . . 1
- 1.2 Installation . . . 1
  - 1.2.1 Install on Linux, Mac OS X . . . 1
  - 1.2.2 Install on Windows . . . 2
- 1.3 Quickstart . . . 2
- 1.4 License . . . 3

2 Documentation index: **5**
- 2.1 User Guide . . . 5
  - 2.1.1 Description . . . 5
  - 2.1.2 Installation . . . 5
  - 2.1.3 Get the source code . . . 6
  - 2.1.4 Dependencies . . . 6
  - 2.1.5 Basic usage . . . 7
- 2.2 The nmrstarlib Tutorial . . . 7
  - 2.2.1 Using nmrstarlib as a library . . . 7
  - 2.2.2 Command Line Interface . . . 15
- 2.3 The nmrstarlib API Reference . . . 18
  - 2.3.1 nmrstarlib.nmrstarlib . . . 18
  - 2.3.2 nmrstarlib.bmrblex . . . 22
  - 2.3.3 nmrstarlib.converter . . . 23
  - 2.3.4 nmrstarlib.csviewer . . . 27
- 2.4 License . . . 28

3 Indices and tables **29**

Python Module Index **31**

Index **33**

CHAPTER

ONE

NMRSTARLIB

The *nmrstarlib* package is a Python library that facilitates reading and writing NMR-STAR formatted files used by the Biological Magnetic Resonance Data Bank (BMRB) for archival of Nuclear Magnetic Resonance (NMR) data.

The *nmrstarlib* package provides facilities to convert NMR-STAR formatted files into their equivalent JSONized (JavaScript Object Notation, an open-standard format that uses human-readable text to transmit data objects consisting of attribute-value pairs) representation and vice versa.

In addition, the nmrstarlib package provides methods to visualize chemical shift data.

The *nmrstarlib* package can be used in several ways:

- As a library for accessing and manipulating data stored in NMR-STAR format files.
- As a command-line tool to convert between NMR-STAR format and its equivalent JSONized NMR-STAR format and also to visualize chemical shift data.

1.1 Links

- nmrstarlib @ GitHub
- nmrstarlib @ PyPI
- Documentation @ ReadTheDocs

1.2 Installation

The *nmrstarlib* package runs under Python 2.7 and Python 3.4+, use pip to install. Starting with Python 3.4 pip is included by default.

1.2.1 Install on Linux, Mac OS X

```
python3 -m pip install nmrstarlib
```

Also make sure that dependencies are installed on the system:

```
python3 -m pip install docopt
python3 -m pip install graphviz
```

graphviz Python library requires a working installation of Graphviz (download page).

1.2.2 Install on Windows

```
py -3 -m pip install nmrstarlib
```

Also make sure that dependencies are installed on the system:

```
py -3 -m pip install docopt
py -3 -m pip install graphviz
```

graphviz Python library requires a working installation of Graphviz (download page).

1.3 Quickstart

Import *nmrstarlib* library and create generator function that will yield *nmrstarlib.nmrstarlib.StarFile* instance(s):

```
>>> from nmrstarlib import nmrstarlib
>>>
>>> # "path": path_to_file / path_to_dir / path_to_archive / bmrb_id / file_url
>>> starfile_gen = nmrstarlib.read_files("path")
>>>
>>> for starfile in starfile_gen:
...     print(starfile.bmrbid)          # print BMRB id of StarFile
...     print(starfile.source)          # print source of StarFile
...     print(list(starfile.keys()))    # print StarFile saveframe categories
>>>
>>> # For example, let's read two files: one using BMRB id and the other one using URL:
>>> starfile_gen = nmrstarlib.read_files("15000", "http://rest.bmrb.wisc.edu/bmrb/NMR-STAR3/18569")
>>>
>>> for starfile in starfile_gen:
...     print("BMRB id:", starfile.bmrbid)
...     print("Source:", starfile.source)
...     print("List of saveframes and comments:", list(starfile.keys()))
BMRB id: 15000
Source: http://rest.bmrb.wisc.edu/bmrb/NMR-STAR3/15000
List of saveframes and comments: ['data', 'comment_0', 'save_entry_information', 'comment_1',
'save_citation_1', 'comment_2', 'save_assembly', 'comment_3', 'save_F5-Phe-cVHP', 'comment_4',
'save_natural_source', 'comment_5', 'save_experimental_source', 'comment_6','save_chem_comp_PHF',
'comment_7', 'comment_8', 'save_unlabeled_sample', 'save_selectively_labeled_sample',
'comment_9', 'save_sample_conditions', 'comment_10', 'save_NMRPipe', 'save_PIPP', 'save_SPARKY',
'save_CYANA', 'save_X-PLOR_NIH', 'comment_11', 'comment_12', 'save_spectrometer_1',
'save_spectrometer_2', 'save_spectrometer_3', 'save_spectrometer_4', 'save_spectrometer_5',
'save_spectrometer_6', 'save_NMR_spectrometer_list', 'comment_13', 'save_experiment_list',
'comment_14', 'comment_15', 'comment_16', 'save_chemical_shift_reference_1', 'comment_17',
'comment_18', 'save_assigned_chem_shift_list_1']
BMRB id: 18569
Source: http://rest.bmrb.wisc.edu/bmrb/NMR-STAR3/18569
List of saveframes and comments: ['data', 'comment_0', 'save_entry_information', 'comment_1',
'save_entry_citation', 'comment_2', 'save_assembly', 'comment_3', 'save_EVH1', 'comment_4',
'save_natural_source', 'comment_5', 'save_experimental_source', 'comment_6', 'comment_7',
'save_sample_1', 'save_sample_2', 'save_sample_3', 'save_sample_4', 'comment_8',
'save_sample_conditions_1', 'save_sample_conditions_2', 'save_sample_conditions_3',
'save_sample_conditions_4', 'comment_9', 'save_AZARA', 'save_xwinnmr', 'save_ANSIG',
'save_CNS', 'comment_10', 'comment_11', 'save_spectrometer_1', 'save_spectrometer_2',
'save_NMR_spectrometer_list', 'comment_12', 'save_experiment_list', 'comment_13',
'comment_14', 'comment_15', 'save_chemical_shift_reference_1', 'comment_16', 'comment_17',
'save_assigned_chem_shift_list_1', 'comment_18', 'save_combined_NOESY_peak_list']
```

Note: Read User Guide and The nmrstarlib Tutorial on ReadTheDocs to learn more and see code examples on using *nmrstarlib* as a library and as a command-line tool.

1.4 License

This package is distributed under the MIT *license*.

CHAPTER

TWO

DOCUMENTATION INDEX:

2.1 User Guide

2.1.1 Description

The nmrstarlib package provides a simple Python interface for parsing and manipulating data stored in NMR-STAR format files used by Biological Magnetic Resonance Data Bank (BMRB) for archival of Nuclear Magnetic Resonance (NMR) experimental data.

Also the nmrstarlib package provides facilities to convert NMR-STAR formatted files into their equivalent JSONized (JavaScript Object Notation, an open-standard format that uses human-readable text to transmit data objects consisting of attribute-value pairs) representation and visa versa.

In addition, the nmrstarlib package provides facilities to visualize assigned chemical shift data.

2.1.2 Installation

The nmrstarlib package runs under Python 2.7 and Python 3.4+. Starting with Python 3.4 pip is included by default. To install system-wide with pip run the following:

Install on Linux, Mac OS X

```
python3 -m pip install nmrstarlib
```

Also make sure that dependencies are installed on the system:

```
python3 -m pip install docopt
python3 -m pip install graphviz
```

graphviz Python library requires a working installation of Graphviz (download page).

Install on Windows

```
py -3 -m pip install nmrstarlib
```

Also make sure that dependencies are installed on the system:

```
py -3 -m pip install docopt
py -3 -m pip install graphviz
```

graphviz Python library requires a working installation of Graphviz (download page).

Install inside virtualenv

For an isolated install, you can run the same inside a virtualenv.

```
$ virtualenv -p /usr/bin/python3 venv  # create virtual environment, use python3 interpreter

$ source venv/bin/activate             # activate virtual environment

$ python3 -m pip install nmrstarlib    # install nmrstarlib as usually

$ deactivate                           # if you are done working in the virtual environment
```

2.1.3 Get the source code

Code is available on GitHub: https://github.com/MoseleyBioinformaticsLab/nmrstarlib

You can either clone the public repository:

```
$ https://github.com/MoseleyBioinformaticsLab/nmrstarlib.git
```

Or, download the tarball and/or zipball:

```
$ curl -OL https://github.com/MoseleyBioinformaticsLab/nmrstarlib/tarball/master

$ curl -OL https://github.com/MoseleyBioinformaticsLab/nmrstarlib/zipball/master
```

Once you have a copy of the source, you can embed it in your own Python package, or install it into your system site-packages easily:

```
$ python3 setup.py install
```

2.1.4 Dependencies

`nmrstarlib` depends on several Python libraries:

- **docopt for creating `nmrstarlib` command-line interface.**
  - To install docopt run the following:

    ```
    python3 -m pip install docopt   # On Linux, Mac OS X
    py -3 -m pip install docopt     # On Windows
    ```

- **graphviz for visualizing assigned chemical shift values.**
  - To install graphviz Python library run the following:

    ```
    python3 -m pip install graphviz   # On Linux, Mac OS X
    py -3 -m pip install graphviz     # On Windows
    ```

  - The only dependency of graphviz Python library is a working installation of Graphviz (download page).

2.1.5 Basic usage

nmrstarlib can be used in several ways:

- As a library for accessing and manipulating data stored in NMR-STAR format files.
  - Create the StarFile generator function that will generate (yield) single StarFile instance at a time.
  - Process each StarFile instance:
    - Process NMR-STAR files in a for-loop one file at a time.
    - Process as an iterator calling the next() built-in function.
    - Convert the generator into a list of StarFile objects.
- As a command-line tool:
  - Convert from NMR-STAR file format into its equivalent JSON file format and vice versa.
  - Visualize (organize) assigned chemical shift values.

Note: Read *The nmrstarlib Tutorial* to learn more and see code examples on using nmrstarlib as a library and as a command-line tool.

2.2 The nmrstarlib Tutorial

The nmrstarlib package provides classes and other facilities for parsing, accessing, and manipulating data stored in NMR-STAR and JSONized NMR-STAR formats. Also, nmrstarlib package provides simple command-line interface.

2.2.1 Using nmrstarlib as a library

Importing nmrstarlib module

If nmrstarlib package is installed on the system the nmrstarlib.nmrstarlib module can be imported:

```
>>> from nmrstarlib import nmrstarlib
```

Constructing StarFile generator

The nmrstarlib module provides read_files() generator function that yields StarFile instances. Constructing StarFile generator is easy - specify path to local NMR-STAR file, directory of NMR-STAR files, archive of NMR-STAR files or BMRB id:

```
>>> from nmrstarlib import nmrstarlib
>>>
>>> single_starfile = nmrstarlib.read_files("bmr18569.str")  # single NMR-STAR file
>>>
>>> starfiles = nmrstarlib.read_files("bmr18569.str", "bmr336.str") # several NMR-STAR files
>>>
>>> dir_starfiles = nmrstarlib.read_files("starfiles_dir")   # directory of NMR-STAR files
>>>
>>> arch_starfiles = nmrstarlib.read_files("starfiles.zip")  # archive of NMR-STAR files
>>>
```

```
>>> url_starfile = nmrstarlib.read_files("18569")           # BMRB id of NMR-STAR file
>>>
```

Processing StarFile generator

The StarFile generator can be processed in several ways:

- Feed it to a for-loop and process one file at a time:

```
>>> for starfile in dir_starfiles:
...     print(starfile.bmrbid)                   # print BMRB id of StarFile
...     print(starfile.source)                   # print source of StarFile
...     for saveframe_name in starfile.keys():   # print saveframe names
...         print(saveframe_name)
>>>
```

Note: Once generator is consumed it becomes empty and needs to be created again.

- Since the StarFile generator behaves like an iterator, we can call the next() built-in function:

```
>>> starfile1 = next(dir_starfiles)
>>> starfile2 = next(dir_starfiles)
>>> ...
```

Note: Once the generator is consumed, StopIteration will be raised.

- Convert the StarFile generator into a list of StarFile objects:

```
>>> starfiles_list = list(dir_starfiles)
>>>
```

Warning: Do not convert the StarFile generator into a list if the generator can yield a large number of files, e.g. several thousand, otherwise it can consume all available memory.

Accessing and manipulating data from a single StarFile

Since StarFile is a Python collections.OrderedDict, data can be accessed and manipulated as with any regular Python dict object using bracket accessors.

- Accessing data in StarFile:

```
>>> list(starfile.keys())  # list StarFile-level keys, i.e. saveframe names
['data', 'save_entry_information', 'save_entry_citation', 'save_assembly',
 'save_EVH1', 'save_natural_source', 'save_experimental_source',
 'save_sample_1', 'save_sample_2', 'save_sample_3', 'save_sample_4',
 'save_sample_conditions_1', 'save_sample_conditions_2',
 'save_sample_conditions_3', 'save_sample_conditions_4', 'save_AZARA',
 'save_xwinnmr', 'save_ANSIG', 'save_CNS', 'save_spectrometer_1',
 'save_spectrometer_2', 'save_NMR_spectrometer_list', 'save_experiment_list',
 'save_chemical_shift_reference_1', 'save_assigned_chem_shift_list_1',
 'save_combined_NOESY_peak_list']
>>>
>>> starfile["data"]
'18569'
>>>
```

```
>>> starfile["save_entry_information"]
OrderedDict([
 ('Entry.Sf_category', 'entry_information'),
 ('Entry.Sf_framecode', 'entry_information'),
 ('Entry.ID', '18569'),
 ('Entry.Title', ';\n13C, 15N and 1H backbone and sidechain assignments\n of the
                  ENA-VASP homology 1 (EVH1) domain of the human
                  vasodilator-stimulated phosphoprotein (VASP)\n;'),
 ('Entry.Type', '.'),
 ('Entry.Version_type', 'original'),
 ('Entry.Submission_date', '2012-07-05'),
 ('Entry.Accession_date', '2012-07-05'), ...
])
>>>
>>> list(starfile["save_entry_information"].keys())  # list saveframe-level keys
['Entry.Sf_category', 'Entry.Sf_framecode', 'Entry.ID', 'Entry.Title',
 'Entry.Type', 'Entry.Version_type', 'Entry.Submission_date',
 'Entry.Accession_date', 'Entry.Last_release_date', 'Entry.Original_release_date',
 'Entry.Origination', 'Entry.NMR_STAR_version', 'Entry.Original_NMR_STAR_version',
 'Entry.Experimental_method', 'Entry.Experimental_method_subtype', 'Entry.Details',
 'Entry.BMRB_internal_directory_name', 'loop_0', 'loop_1', 'loop_2', 'loop_3', 'loop_4']
>>>
>>> starfile["save_entry_information"]["Entry.Submission_date"]
'2012-07-05'
>>>
>>> starfile["save_entry_information"]["loop_0"]
(['Entry_author.Ordinal', 'Entry_author.Given_name', 'Entry_author.Family_name',
  'Entry_author.First_initial', 'Entry_author.Middle_initials',
  'Entry_author.Family_title', 'Entry_author.Entry_ID'],
  [OrderedDict([('Entry_author.Ordinal', '1'),
                ('Entry_author.Given_name', 'Linda'),
                ('Entry_author.Family_name', 'Ball'),
                ('Entry_author.First_initial', '.'),
                ('Entry_author.Middle_initials', 'J.'),
                ('Entry_author.Family_title', '.'),
                ('Entry_author.Entry_ID', '18569')]),
   OrderedDict([('Entry_author.Ordinal', '2'),
                ('Entry_author.Given_name', 'Schmieder'),
                ('Entry_author.Family_name', 'Peter'),
                ('Entry_author.First_initial', '.'),
                ('Entry_author.Middle_initials', '.'),
                ('Entry_author.Family_title', '.'),
                ('Entry_author.Entry_ID', '18569')])
])
>>>
>>> starfile["save_entry_information"]["loop_0"][0]  # list loop-level keys
['Entry_author.Ordinal', 'Entry_author.Given_name', 'Entry_author.Family_name',
'Entry_author.First_initial', 'Entry_author.Middle_initials',
'Entry_author.Family_title', 'Entry_author.Entry_ID']
>>>
>>> # loop values is a list of dictionaries:
>>> starfile["save_entry_information"]["loop_0"][1]
[OrderedDict([('Entry_author.Ordinal', '1'),
              ('Entry_author.Given_name', 'Linda'),
              ('Entry_author.Family_name', 'Ball'),
              ('Entry_author.First_initial', '.'),
              ('Entry_author.Middle_initials', 'J.'),
              ('Entry_author.Family_title', '.'),
```

```
                ('Entry_author.Entry_ID', '18569')]),
 OrderedDict([('Entry_author.Ordinal', '2'),
              ('Entry_author.Given_name', 'Schmieder'),
              ('Entry_author.Family_name', 'Peter'),
              ('Entry_author.First_initial', '.'),
              ('Entry_author.Middle_initials', '.'),
              ('Entry_author.Family_title', '.'),
              ('Entry_author.Entry_ID', '18569')])]
>>>
>>> # every loop entry is accessed by index:
>>> starfile["save_entry_information"]["loop_0"].[1][0]["Entry_author.Family_name"]
'Ball'
>>> starfile["save_entry_information"]["loop_0"].[1][1]["Entry_author.Family_name"]
'Peter'
```

- Manipulating data in a `StarFile` is easy - access data using bracket accessors and set a new value:

```
>>> starfile["data"]
'18569'
>>>
>>> starfile["data"] = "18569_modified"
'18569_modified'
>>>
>>> # change submission date
>>> starfile["save_entry_information"]["Entry.Submission_date"]
'2012-07-05'
>>>
>>> starfile["save_entry_information"]["Entry.Submission_date"] = "2015-07-05"
'2015-07-05'
>>>
```

- Printing a `StarFile` and its components (*saveframe* and *loop* data):

```
>>> starfile.print_starfile(file_format="nmrstar")
data_18569
save_entry_information
    _Entry.Sf_category     entry_information
    _Entry.Sf_framecode    entry_information
    _Entry.ID      18569
...
>>>
>>> starfile.print_starfile(file_format="json")
{
 "data": "18569",
 "save_entry_information": {
     "Entry.Sf_category": "entry_information",
     "Entry.Sf_framecode": "entry_information",
     "Entry.ID": "18569",
 ...
}
>>>
>>> starfile.print_saveframe("save_entry_information", file_format="nmrstar")
_Entry.Sf_category         entry_information
_Entry.Sf_framecode        entry_information
_Entry.ID  18569
_Entry.Title
;
13C, 15N and 1H backbone and sidechain assignments of the
ENA-VASP homology 1 (EVH1) domain of the human
```

```
vasodilator-stimulated phosphoprotein (VASP)
;
_Entry.Type        .
_Entry.Version_type        original
_Entry.Submission_date     2012-07-05
_Entry.Accession_date      2012-07-05
_Entry.Last_release_date   2012-07-18
_Entry.Original_release_date       2012-07-18
_Entry.Origination         author
_Entry.NMR_STAR_version    3.1.1.61
_Entry.Original_NMR_STAR_version   3.1
_Entry.Experimental_method         NMR
_Entry.Experimental_method_subtype         solution
_Entry.Details     'ANSIG v3.3 exported crosspeaks file'
_Entry.BMRB_internal_directory_name        .
...
>>>
>>> starfile.print_saveframe("save_entry_information", file_format="json")
{
    "Entry.Sf_category": "entry_information",
    "Entry.Sf_framecode": "entry_information",
    "Entry.ID": "18569",
    "Entry.Title": ";\n13C, 15N and 1H backbone and sidechain assignments of the
                     ENA-VASP homology 1 (EVH1) domain of the human
                     vasodilator-stimulated phosphoprotein (VASP)\n;",
    "Entry.Type": ".",
    "Entry.Version_type": "original",
    "Entry.Submission_date": "2012-07-05",
    "Entry.Accession_date": "2012-07-05",
    "Entry.Last_release_date": "2012-07-18",
    "Entry.Original_release_date": "2012-07-18",
    "Entry.Origination": "author",
    "Entry.NMR_STAR_version": "3.1.1.61",
    "Entry.Original_NMR_STAR_version": "3.1",
    "Entry.Experimental_method": "NMR",
    "Entry.Experimental_method_subtype": "solution",
    "Entry.Details": "'ANSIG v3.3 exported crosspeaks file'",
    "Entry.BMRB_internal_directory_name": ".",
    ...
}
>>>
>>> starfile.print_loop("save_entry_information", "loop_1", file_format="nmrstar")
_Data_set.Type
_Data_set.Count
_Data_set.Entry_ID
assigned_chemical_shifts 1 18569
spectral_peak_list 1 18569
>>>
>>> starfile.print_loop("save_entry_information", "loop_1", file_format="json")
[
    [
        "Data_set.Type",
        "Data_set.Count",
        "Data_set.Entry_ID"
    ],
    [
        {
            "Data_set.Type": "assigned_chemical_shifts",
```

```
                "Data_set.Count": "1",
                "Data_set.Entry_ID": "18569"
            },
            {
                "Data_set.Type": "spectral_peak_list",
                "Data_set.Count": "1",
                "Data_set.Entry_ID": "18569"
            }
        ]
    ]
    >>>
```

- Accessing chemical shift data:

Chemical shift data can be accessed using bracket accessors as described above using a *saveframe* name and *loop* name:

```
>>> starfile["save_assigned_chem_shift_list_1"]["loop_1"][0]
['Atom_chem_shift.ID', 'Atom_chem_shift.Assembly_atom_ID',
 'Atom_chem_shift.Entity_assembly_ID', 'Atom_chem_shift.Entity_ID',
 'Atom_chem_shift.Comp_index_ID', 'Atom_chem_shift.Seq_ID',
 'Atom_chem_shift.Comp_ID', 'Atom_chem_shift.Atom_ID',
 'Atom_chem_shift.Atom_type', 'Atom_chem_shift.Atom_isotope_number',
 'Atom_chem_shift.Val', 'Atom_chem_shift.Val_err',
 'Atom_chem_shift.Assign_fig_of_merit', 'Atom_chem_shift.Ambiguity_code',
 'Atom_chem_shift.Occupancy', 'Atom_chem_shift.Resonance_ID',
 'Atom_chem_shift.Auth_entity_assembly_ID', 'Atom_chem_shift.Auth_asym_ID',
 'Atom_chem_shift.Auth_seq_ID', 'Atom_chem_shift.Auth_comp_ID',
 'Atom_chem_shift.Auth_atom_ID', 'Atom_chem_shift.Details',
 'Atom_chem_shift.Entry_ID', 'Atom_chem_shift.Assigned_chem_shift_list_ID']
>>>
>>> starfile["save_assigned_chem_shift_list_1"]["loop_1"][1][0]["Atom_chem_shift.Seq_ID"]
'1'
>>> starfile["save_assigned_chem_shift_list_1"]["loop_1"][1][0]["Atom_chem_shift.Comp_ID"]
'MET'
>>> starfile["save_assigned_chem_shift_list_1"]["loop_1"][1][0]["Atom_chem_shift.Atom_ID"]
'H'
>>> starfile["save_assigned_chem_shift_list_1"]["loop_1"][1][0]["Atom_chem_shift.Val"]
'8.55'
>>> starfile["save_assigned_chem_shift_list_1"]["loop_1"][1][1]["Atom_chem_shift.Atom_ID"]
'HA'
>>> starfile["save_assigned_chem_shift_list_1"]["loop_1"][1][1]["Atom_chem_shift.Val"]
'4.548'
>>> starfile["save_assigned_chem_shift_list_1"]["loop_1"][1][2]["Atom_chem_shift.Atom_ID"]
'HB2'
>>> starfile["save_assigned_chem_shift_list_1"]["loop_1"][1][2]["Atom_chem_shift.Val"]
'1.994'
>>>
```

Also the `StarFile` class provides a `chem_shifts_by_residue()` method that organizes chemical shits into `list` of `collections.OrderedDict` data structures (*keys* - sequence id, *values* - chemical shift data) - one for each protein chain, if multiple chains are present within the file:

```
>>> starfile.chem_shifts_by_residue()
[OrderedDict([
    ('1', OrderedDict([('AA3Code', 'MET'),
                       ('Seq_ID', '1'),
                       ('H', '8.55'),
                       ('HA', '4.548'),
```

```
                                ('HB2', '1.994'),
                                ('HB3', '2.118'),
                                ('CA', '55.489'),
                                ('CB', '32.848'),
                                ('N', '122.221')])),
        ('2', OrderedDict([('AA3Code', 'SER'),
                                ('Seq_ID', '2'),
                                ('H', '8.225'),
                                ('HA', '4.420'),
                                ('HB2', '3.805'),
                                ('HB3', '3.857'),
                                ('CA', '58.593'),
                                ('CB', '64.057'),
                                ('N', '117.197')])),
        ('3', OrderedDict([('AA3Code', 'GLU'),
                                ('Seq_ID', '3'),
                                ('H', '8.002'),
                                ('HA', '4.848'),
                                ('HB2', '1.852'),
                                ('HB3', '1.963'),
                                ('HG2', '1.981'),
                                ('HG3', '2.191'),
                                ('CA', '55.651'),
                                ('CB', '32.952'),
                                ('CG', '37.425'),
                                ('N', '119.833')])), ...
    ...
    ]
    >>>
    >>> starfile.chem_shifts_by_residue(amino_acids=["SER"], atoms=["CA", "CB"])
    [OrderedDict([
        ('2', OrderedDict([('AA3Code', 'SER'),
                                ('Seq_ID', '2'),
                                ('CA', '58.593'),
                                ('CB', '64.057')])),
        ('8', OrderedDict([('AA3Code', 'SER'),
                                ('Seq_ID', '8'),
                                ('CA', '57.456'),
                                ('CB', '64.863')])),
        ('9', OrderedDict([('AA3Code', 'SER'),
                                ('Seq_ID', '9'),
                                ('CA', '57.852'),
                                ('CB', '67.332')])),
        ('34', OrderedDict([('AA3Code', 'SER'),
                                 ('Seq_ID', '34'),
                                 ('CA', '59.113'),
                                 ('CB', '66.248')])),
        ('46', OrderedDict([('AA3Code', 'SER'),
                                 ('Seq_ID', '46'),
                                 ('CA', '55.939'),
                                 ('CB', '66.829')])),
        ('95', OrderedDict([('AA3Code', 'SER'),
                                 ('Seq_ID', '95'),
                                 ('CA', '57.013'),
                                 ('CB', '66.501')])),
        ('108', OrderedDict([('AA3Code', 'SER'),
                                  ('Seq_ID', '108'),
                                  ('CA', '61.617'),
```

```
                                  ('CB', '62.493')]))])
]
>>>
```

Writing data from a StarFile object into a file

Data from `StarFile` can be written into file in original NMR-STAR format or in equivalent JSON format using `write()`:

- Writing into a NMR-STAR formatted file:

```
>>> with open("bmr18569_modified.str", "w") as outfile:
...     starfile.write(outfile, file_format="nmrstar")
>>>
```

- Writing into a JSONized NMR-STAR formatted file:

```
>>> with open("bmr18569_modified.json", "w") as outfile:
...     starfile.write(outfile, file_format="json")
>>>
```

Converting NMR-STAR files

NMR-STAR files can be converted between the NMR-STAR file format and a JSONized NMR-STAR file format using the `nmrstarlib.converter` module.

- Converting from the NMR-STAR file format into its equivalent JSON file format:

```
>>> from nmrstarlib.converter import Converter
>>>
>>> # Using valid BMRB id to access file from URL: from_path="18569"
>>> converter = Converter(from_path="18569", to_path="bmr18569.json",
...                       from_format="nmrstar", to_format="json")
>>> converter.convert()
>>>
```

- Converting from JSON file format into its equivalent NMR-STAR file format:

```
>>> from nmrstarlib.converter import Converter
>>>
>>> converter = Converter(from_path="bmr18569.json", to_path="bmr18569.str",
...                       from_format="json", to_format="nmrstar")
>>> converter.convert()
>>>
```

Note: See `nmrstarlib.converter` for full list of available conversions.

Visualizing chemical shifts values

Chemical shifts values can be visualized using the `nmrstarlib.csviewer` Chemical Shifts Viewer module.

```
>>> from nmrstarlib.csviewer import CSViewer
>>>
>>> csviewer = CSViewer(from_path="18569", filename="18569_chem_shifts_all", csview_format="png")
>>> csviewer.csview(view=True)
```

```
>>>
>>> csviewer = CSViewer(from_path="18569", amino_acids=["SER", "THR"], atoms=["CA", "CB"],
...                     filename="18569_chem_shifts_SER_THR_CA_CB", csview_format="png")
>>> csviewer.csview(view=True)  # open in a default image viewer or pdf viewer
>>> csviewer.csview(view=False) # save output file in current working directory
>>>
```

`nmrstarlib.csviewer` output example:

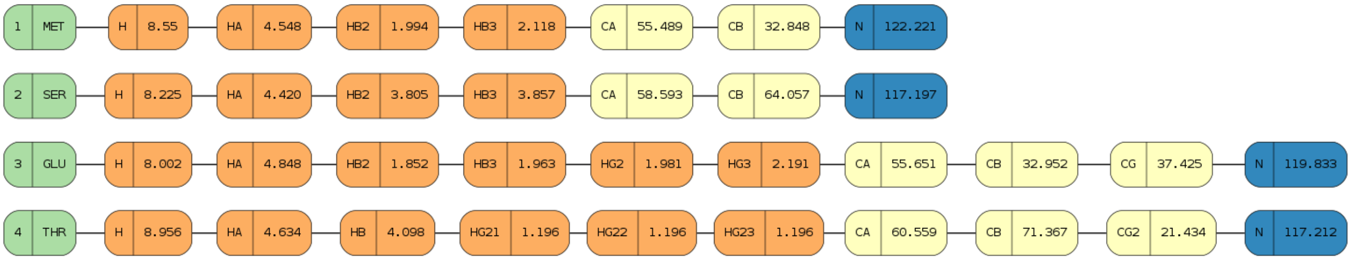

2.2.2 Command Line Interface

Command Line Interface functionality:

- Convert from NMR-STAR file format into its equivalent JSON file format and vice versa.
- Visualize assigned chemical shift values.

```
nmrstarlib command-line interface

Usage:
    nmrstarlib -h | --help
    nmrstarlib --version
    nmrstarlib convert (<from_path> <to_path>) [--from_format=<format>]
                                                [--to_format=<format>]
                                                [--bmrb_url=<url>]
                                                [--nmrstar_version=<version>]
                                                [--verbose]

    nmrstarlib csview <starfile_path> [--amino_acids=<aa>]
                                      [--atoms=<at>]
                                      [--csview_outfile=<path>]
                                      [--csview_format=<format>]
                                      [--nmrstar_version=<version>]
                                      [--verbose]

Options:
    -h, --help                    Show this screen.
    --version                     Show version.
    --verbose                     Print what files are processing.
    --from_format=<format>        Input file format, available formats:
                                  nmrstar, json [default: nmrstar].
    --to_format=<format>          Output file format, available formats:
                                  nmrstar, json [default: json].
    --nmrstar_version=<version>   Version of NMR-STAR format to use, available:
                                  3, 2 [default: 3].
    --bmrb_url=<url>              URL to BMRB REST interface
                                  [default: http://rest.bmrb.wisc.edu/bmrb/NMR-STAR3/].
```

```
--amino_acids=<aa>          Comma-separated amino acid three-letter codes.
--atoms=<at>                Comma-separated BMRB atom codes.
--csview_outfile=<path>     Where to save chemical shifts table.
--csview_format=<format>    Format to which save chamical shift table
                            [default: svg].
```

Converting NMR-STAR files in bulk

One-to-one file conversions

- Convert from a local file in NMR-STAR format to a local file in JSON format:

```
$ python3 -m nmrstarlib convert bmr18569.str bmr18569.json \
          --from_format=nmrstar --to_format=json
```

- Convert from a local file in JSON format to a local file in NMR-STAR format:

```
$ python3 -m nmrstarlib convert bmr18569.json bmr18569.str \
          --from_format=json --to_format=nmrstar
```

- Convert from a compressed local file in NMR-STAR format to a compressed local file in JSON format:

```
$ python3 -m nmrstarlib convert bmr18569.str.gz bmr18569.json.gz \
          --from_format=nmrstar --to_format=json
```

- Convert from a compressed local file in JSON format to a compressed local file in NMR-STAR format:

```
$ python3 -m nmrstarlib convert bmr18569.json.gz bmr18569.str.gz \
          --from_format=json --to_format=nmrstar
```

- Convert from a uncompressed URL file in NMR-STAR format to a compressed local file in JSON format:

```
$ python3 -m nmrstarlib convert 18569 bmr18569.json.bz2 \
          --from_format=nmrstar --to_format=json
```

Note: See `nmrstarlib.converter` for full list of available conversions.

Many-to-many files conversions

- Convert from a directory of files in NMR-STAR format to a directory of files in JSON format:

```
$ python3 -m nmrstarlib convert starfiles_dir_nmrstar starfiles_dir_json \
          --from_format=nmrstar --to_format=json
```

- Convert from a directory of files in JSON format to a directory of files in NMR-STAR format:

```
$ python3 -m nmrstarlib convert starfiles_dir_json starfiles_dir_nmrstar \
          --from_format=json --to_format=nmrstar
```

- Convert from a directory of files in NMR-STAR format to a zip archive of files in JSON format:

```
$ python3 -m nmrstarlib convert starfiles_dir_nmrstar starfiles_json.zip \
          --from_format=nmrstar --to_format=json
```

- Convert from a compressed tar archive of files in JSON format to a directory of files in NMR-STAR format:

```
$ python3 -m nmrstarlib convert starfiles_json.tar.gz starfiles_dir_nmrstar \
          --from_format=json --to_format=nmrstar
```

- Convert from a zip archive of files in NMR-STAR format to a compressed tar archive of files in JSON format:

```
$ python3 -m nmrstarlib convert starfiles_nmrstar.zip starfile_json.tar.bz2 \
          --from_format=nmrstar --to_format=json
```

Note: See `nmrstarlib.converter` for full list of available conversions.

Visualizing chemical shift values

- Visualize chemical shift values for the entire sequence:

```
$ python3 -m nmrstarlib csview 18569 \
          --csview_outfile=18569_chem_shifts_all --csview_format=png
```

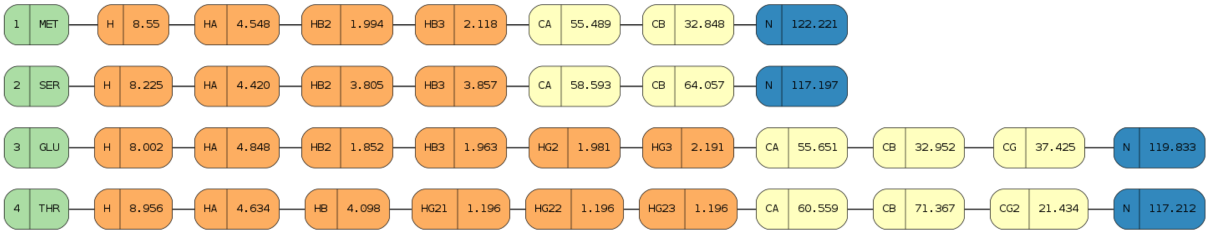

- Visualize *CA*, *CB*, *CG*, and *CG2* chemical shift values for *GLU* and *THR* amino acid residues:

```
$ python3 -m nmrstarlib csview 18569 \
          --amino_acids=GLU,THR --atoms=CA,CB,CG,CG2 \
          --csview_outfile=18569_chem_shifts_GLU_THR_CA_CB_CG_CG2 \
          --csview_format=png
```

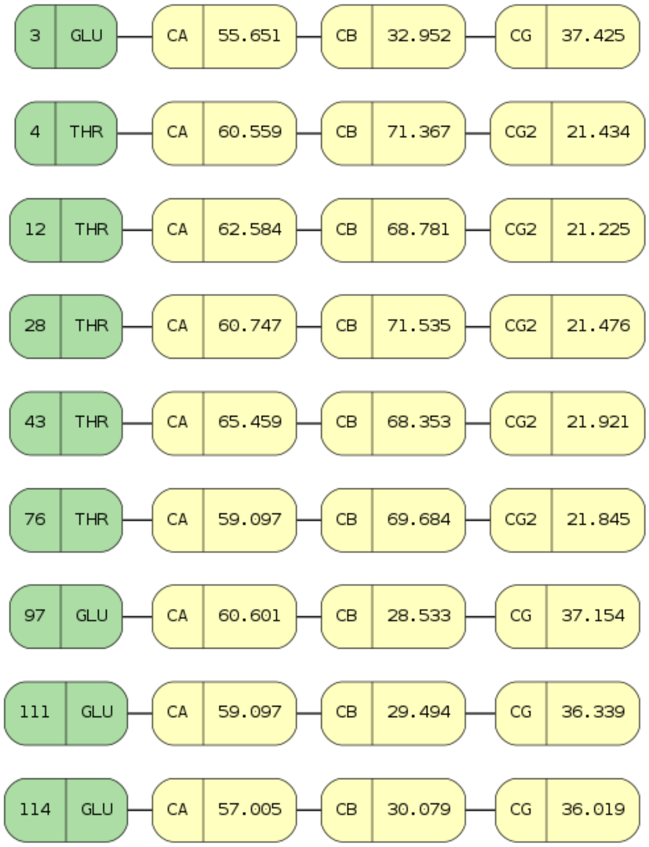

2.3 The nmrstarlib API Reference

Routines for working with BMRB NMR-STAR format files.

This package includes the following modules:

nmrstarlib This module provides the `StarFile` class which is a python dictionary representation of a BMRB NMR-STAR file. Data can be accessed directly from the `StarFile` instance using bracket accessors. The `nmrstarlib` module relies on the `bmrblex` module for processing of tokens.

bmrblex This module provides the `bmrblex()` generator that is responsible for the syntax analysis of BMRB NMR-STAR files, processing word, number, single quoted, double quoted, multiline quoted BMRB tokens.

converter This module provides the `Converter` class that is responsible for the conversion between NMR-STAR formated files and an equivalent JSONized file format.

csviewer This module provides the `CSViewer` class that visualizes chemical shift values using the Graphviz (http://www.graphviz.org/) DOT Languge description and provides code example for utilizing the library.

2.3.1 nmrstarlib.nmrstarlib

This module provides the `StarFile` class that stores the data from a single NMR-STAR file in the form of an `OrderedDict`. Data can be accessed directly from the `StarFile` instance using bracket accessors.

The NMR-STAR format is a hierachical dictionary containing data on NMR experiments. The data is divided into a series of "saveframes" which each contain a number of key-value pairs and "loops".

Each saveframe has a unique name, which is used as the key in the dictionary, corresponding to another dictionary containing the information in the saveframe. Since loops in NMR-Star format do not have names, the keys for them inside the saveframe dictionary are simply loop_0, loop_1, etc.

class `nmrstarlib.nmrstarlib.`**`StarFile`**(*source='', frame_categories=None, *args, **kwds*)
StarFile class that stores the data from a single NMR-STAR file in the form of an `OrderedDict`.

`__init__`(*source='', frame_categories=None, *args, **kwds*)
StarFile initializer. Leave *frame_categories* as `None` to read everything. Otherwise it can be a list of saveframe categories to read, skipping the rest.

Parameters

- **source** (*str*) – Source *StarFile* instance was created from - local file or URL address.
- **frame_categories** (*list*) – List of saveframe names.

`read`(*filehandle*)
Read data into a `StarFile` instance.

Parameters **filehandle** (`io.TextIOWrapper`, `gzip.GzipFile`, `bz2.BZ2File`, `zipfile.ZipFile`) – file-like object.

Returns None

Return type `None`

`write`(*filehandle, file_format*)
Write `StarFile` data into file.

Parameters

- **filehandle** (`io.TextIOWrapper`) – file-like object.
- **file_format** (*str*) – Format to use to write data: *nmrstar* or *json*.

Returns None

Return type `None`

`writestr`(*file_format*)
Write `StarFile` data into string.

Parameters **file_format** (*str*) – Format to use to write data: *nmrstar* or *json*.

Returns String representing the `StarFile` instance.

Return type `str`

`_build_starfile`(*nmrstar_str*)
Build `StarFile` object.

Parameters **nmrstar_str** (`str` or `bytes`) – NMR-STAR-formatted string.

Returns instance of `StarFile`.

Return type `StarFile`

`_build_saveframe`(*lexer*)
Build NMR-STAR file saveframe.

Parameters **lexer** (`bmrblex()`) – instance of the BMRB lexical analyzer.

Returns Saveframe dictionary.

Return type `collections.OrderedDict`

_build_loop (*lexer*)
Build saveframe loop.

Parameters **lexer** (`bmrblex()`) – instance of BMRB lexical analyzer.

Returns Fields and values of the loop.

Return type `tuple`

print_starfile (*f=<_io.TextIOWrapper name='<stdout>' mode='w' encoding='UTF-8'>, file_format='nmrstar', tw=3*)
Print `StarFile` into a file or stdout.

Parameters

- **f** (*io.StringIO*) – writable file-like stream.
- **file_format** (*str*) – Format to use: *nmrstar* or *json*.
- **tw** (*int*) – Tab width.

Returns None

Return type `None`

print_saveframe (*sf, f=<_io.TextIOWrapper name='<stdout>' mode='w' encoding='UTF-8'>, file_format='nmrstar', tw=3*)
Print saveframe into a file or stdout. We need to keep track of how far over everything is tabbed. The "tab width" variable tw does this for us.

Parameters

- **sf** (*str*) – Saveframe name.
- **f** (*io.StringIO*) – writable file-like stream.
- **file_format** (*str*) – Format to use: *nmrstar* or *json*.
- **tw** (*int*) – Tab width.

Returns None

Return type `None`

print_loop (*sf, sftag, f=<_io.TextIOWrapper name='<stdout>' mode='w' encoding='UTF-8'>, file_format='nmrstar', tw=3*)
Print loop into a file or stdout.

Parameters

- **sf** (*str*) – Saveframe name.
- **sftag** (*str*) – Saveframe tag, i.e. field name.
- **f** (*io.StringIO*) – writable file-like stream.
- **file_format** (*str*) – Format to use: *nmrstar* or *json*.
- **tw** (*int*) – Tab width.

Returns None

Return type `None`

_to_json ()
Save `StarFile` into JSON string.

Returns JSON string.

Return type `str`

_to_nmrstar()

Save `StarFile` NMR-STAR format string.

Returns NMR-STAR string.

Return type `str`

_skip_saveframe(*lexer*)

Skip entire saveframe - keep emitting tokens until the end of saveframe.

Parameters **lexer** (`bmrblex`) – instance of the BMRB lexical analyzer class.

Returns None

Return type `None`

static _is_nmrstar(*string*)

Test if input string is in NMR-STAR format.

Parameters **string** (`str` or `bytes`) – Input string.

Returns Input string if in NMR-STAR format or False otherwise.

Return type `str` or `False`

static _is_json(*string*)

Test if input string is in JSON format.

Parameters **string** (`str` or `bytes`) – Input string.

Returns Input string if in JSON format or False otherwise.

Return type `str` or `False`

chem_shifts_by_residue(*amino_acids=None*, *atoms=None*, *nmrstar_version='3'*)

Organize chemical shifts by amino acid residue.

Parameters

- **amino_acids** (*list*) – List of amino_acids three-letter codes.
- **atoms** (*list*) – List of BMRB atom type codes.
- **nmrstar_version** (*str*) – Version of NMR-STAR format to use for look up chemichal shifts loop.

Returns List of OrderedDict per each chain

Return type `list` of `collections.OrderedDict`

nmrstarlib.nmrstarlib.**update_constants**(*filehandle*)

Update constants related to NMR-STAR format, e.g. field names.

Parameters **filehandle** (`io.TextIOWrapper`) – JSON file that contains information about NMR-STAR format.

Returns None

Return type `None`

nmrstarlib.nmrstarlib.**_generate_filenames**(*sources*)

Generate filenames.

Parameters **sources** (*tuple*) – Sequence of strings representing path to file(s).

Returns Path to file(s).

Return type `str`

`nmrstarlib.nmrstarlib.`**`_generate_handles`**(*filenames*)

Open a sequence of filenames one at time producing file objects. The file is closed immediately when proceeding to the next iteration.

Parameters **filenames** (*generator*) – Generator object that yields the path to each file, one at a time.

Returns Filehandle to be processed into a `StarFile` instance.

`nmrstarlib.nmrstarlib.`**`read_files`**(**sources*)

Construct a generator that yields `StarFile` instances.

Parameters **sources** – One or more strings representing path to file(s).

Returns `StarFile` instance(s).

Return type `StarFile`

class `nmrstarlib.nmrstarlib.`**`GenericFilePath`**(*path*)

GenericFilePath class knows how to open local files or files over URL.

`__init__`(*path*)

Initialize path.

Parameters **path** (*str*) – String representing a path to local file(s) or valid URL address of file(s).

`open`()

Generator that opens and yields filehandles using appropriate facilities: test if path represents a local file or file over URL, if file is compressed or not.

Returns Filehandle to be processed into a `StarFile` instance.

static **`is_compressed`**(*path*)

Test if path represents compressed file(s).

Parameters **path** (*str*) – Path to file(s).

Returns String specifying compression type if compressed, "" otherwise.

Return type `str`

static **`is_url`**(*path*)

Test if path represents a valid URL.

Parameters **path** (*str*) – Path to file.

Returns True if path is valid url string, False otherwise.

Return type `True` or `False`

`__weakref__`

list of weak references to the object (if defined)

2.3.2 nmrstarlib.bmrblex

This module provides `bmrblex()` lexical analyzer for BMRB NMR-STAR format syntax. It is implemented as Python generator-based state machine which generates (yields) token one at a time when `next()` is invoked on `bmrblex()` instance.

Simplified description of parsing rules:

- Each word or number separated by whitespace characters is a separate BMRB token.
- Each single quoted (‘) string is a separate BMRB token, it should start with a single quote (‘) and end with a single quote *always* followed by whitespace character(s).
- Each double quoted (”) string is a separate BMRB token, it should start with a double quote (”) and end with a double quote *always* followed by whitespace character(s).
- Single quoted and double quoted strings have to be processed separately.
- Single quoted and double quoted strings are processed one character at a time.
- Multiline strings start with a semicolon *always* followed by new line character and ending with a semicolon *always* followed by whitespace character(s).
- Multiline strings are processed one line at a time.

Note:

- For a full description of NMR-STAR file format, see official documentation: http://www.bmrb.wisc.edu/dictionary/
- For a concise description of the NMR-STAR file format grammar see: https://github.com/mattfenwick/NMRPyStar#nmr-star-grammar

---

`nmrstarlib.bmrblex.`**`bmrblex`**`(`*text*`)`

A lexical analyzer for the BMRB NMR-STAR format syntax.

Parameters **text** (`str` or `bytes`) – Input text.

Returns Current token.

Return type `str`

2.3.3 nmrstarlib.converter

This module provides functionality for converting between the BMRB NMR-STAR format and its equivalent JSONized NMR-STAR format.

The following conversions are possible:

Local files:

- **One-to-one file conversions:**
  - textfile - to - textfile
  - textfile - to - textfile.gz
  - textfile - to - textfile.bz2
  - textfile.gz - to - textfile
  - textfile.gz - to - textfile.gz
  - textfile.gz - to - textfile.bz2
  - textfile.bz2 - to - textfile
  - textfile.bz2 - to - textfile.gz
  - textfile.bz2 - to - textfile.bz2

- textfile / textfile.gz / textfile.bz2 - to - textfile.zip / textfile.tar / textfile.tar.gz / textfile.tar.bz2 (TypeError: One-to-many conversion)

- **Many-to-many files conversions:**
  - **Directories:**
    * directory - to - directory
    * directory - to - directory.zip
    * directory - to - directory.tar
    * directory - to - directory.tar.bz2
    * directory - to - directory.tar.gz
    * directory - to - directory.gz / directory.bz2 (TypeError: Many-to-one conversion)
  - **Zipfiles:**
    * zipfile.zip - to - directory
    * zipfile.zip - to - zipfile.zip
    * zipfile.zip - to - tarfile.tar
    * zipfile.zip - to - tarfile.tar.gz
    * zipfile.zip - to - tarfile.tar.bz2
    * zipfile.zip - to - directory.gz / directory.bz2 (TypeError: Many-to-one conversion)
  - **Tarfiles:**
    * tarfile.tar - to - directory
    * tarfile.tar - to - zipfile.zip
    * tarfile.tar - to - tarfile.tar
    * tarfile.tar - to - tarfile.tar.gz
    * tarfile.tar - to - tarfile.tar.bz2
    * tarfile.tar - to - directory.gz / directory.bz2 (TypeError: Many-to-one conversion)
    * tarfile.tar.gz - to - directory
    * tarfile.tar.gz - to - zipfile.zip
    * tarfile.tar.gz - to - tarfile.tar
    * tarfile.tar.gz - to - tarfile.tar.gz
    * tarfile.tar.gz - to - tarfile.tar.bz2
    * tarfile.tar.gz - to - directory.gz / directory.bz2 (TypeError: Many-to-one conversion)
    * tarfile.tar.bz2 - to - directory
    * tarfile.tar.bz2 - to - zipfile.zip
    * tarfile.tar.bz2 - to - tarfile.tar
    * tarfile.tar.bz2 - to - tarfile.tar.gz
    * tarfile.tar.bz2 - to - tarfile.tar.bz2
    * tarfile.tar.bz2 - to - directory.gz / directory.bz2 (TypeError: Many-to-one conversion)

URL files:

- **One-to-one file conversions:**
  - bmrbid - to - textfile
  - bmrbid - to - textfile.gz
  - bmrbid - to - textfile.bz2
  - bmrbid - to - textfile.zip / textfile.tar / textfile.tar.gz / textfile.tar.bz2 (TypeError: One-to-many conversion)
  - textfileurl - to - textfile
  - textfileurl - to - textfile.gz
  - textfileurl - to - textfile.bz2
  - textfileurl.gz - to - textfile
  - textfileurl.gz - to - textfile.gz
  - textfileurl.gz - to - textfile.bz2
  - textfileurl.bz2 - to - textfile
  - textfileurl.bz2 - to - textfile.gz
  - textfileurl.bz2 - to - textfile.bz2
  - textfileurl / textfileurl.gz / textfileurl.bz2 - to - textfile.zip / textfile.tar / textfile.tar.gz / textfile.tar.bz2 (TypeError: One-to-many conversion)
- **Many-to-many files conversions:**
  - **Zipfiles:**
    * zipfileurl.zip - to - directory
    * zipfileurl.zip - to - zipfile.zip
    * zipfileurl.zip - to - tarfile.tar
    * zipfileurl.zip - to - tarfile.tar.gz
    * zipfileurl.zip - to - tarfile.tar.bz2
    * zipfileurl.zip - to - directory.gz / directory.bz2 (TypeError: Many-to-one conversion)
  - **Tarfiles:**
    * tarfileurl.tar - to - directory
    * tarfileurl.tar - to - zipfile.zip
    * tarfileurl.tar - to - tarfile.tar
    * tarfileurl.tar - to - tarfile.tar.gz
    * tarfileurl.tar - to - tarfile.tar.bz2
    * tarfileurl.tar - to - directory.gz / directory.bz2 (TypeError: Many-to-one conversion)
    * tarfileurl.tar.gz - to - directory
    * tarfileurl.tar.gz - to - zipfile.zip
    * tarfileurl.tar.gz - to - tarfile.tar
    * tarfileurl.tar.gz - to - tarfile.tar.gz

* tarfileurl.tar.gz - to - tarfile.tar.bz2
* tarfileurl.tar.gz - to - directory.gz / directory.bz2 (TypeError: Many-to-one conversion)
* tarfileurl.tar.bz2 - to - directory
* tarfileurl.tar.bz2 - to - zipfile.zip
* tarfileurl.tar.bz2 - to - tarfile.tar
* tarfileurl.tar.bz2 - to - tarfile.tar.gz
* tarfileurl.tar.bz2 - to - tarfile.tar.bz2
* tarfileurl.tar.bz2 - to - directory.gz / directory.bz2 (TypeError: Many-to-one conversion)

class `nmrstarlib.converter.`**`Converter`**(*from_path*, *to_path*, *from_format='nmrstar'*, *to_format='json'*)

Converter class to convert BMRB NMR-STAR files from NMR-STAR to JSON or from JSON to NMR-STAR format.

`__init__`(*from_path*, *to_path*, *from_format='nmrstar'*, *to_format='json'*)

Converter initializer.

Parameters

- **from_path** (*str*) – Path to input file(s).
- **to_path** (*str*) – Path to output file(s).
- **from_format** (*str*) – Input format: *nmrstar* or *json*.
- **to_format** (*str*) – Output format: *nmrstar* or *json*.

Returns None

Return type `None`

`convert`()

Convert file(s) from NMR-STAR format to JSON format or from JSON format to NMR-STAR format.

Returns None

Return type `None`

`_many_to_many`()

Perform many-to-many files conversion.

Returns None

Return type `None`

`_one_to_one`()

Perform one-to-one file conversion.

Returns None

Return type `None`

`_to_dir`()

Convert files to directory.

Returns None

Return type `None`

`_to_zipfile`()

Convert files to zip archive.

Returns None

Return type None

_to_tarfile()
Convert files to tar archive.

Returns None

Return type None

_to_bz2file()
Convert file to bz2-compressed file.

Returns None

Return type None

_to_gzipfile()
Convert file to gzip-compressed file.

Returns None

Return type None

_to_textfile()
Convert file to regular text file.

Returns None

Return type None

_outputpath(*inputpath*, *archive=False*)
Construct an output path string from an input path string.

Parameters **inputpath** (*str*) – Input path string.

Returns Output path string.

Return type str

__weakref__
list of weak references to the object (if defined)

2.3.4 nmrstarlib.csviewer

This module provides the CSViewer class - Chemical Shifts Viewer that visualizes chemical shifts values.

class nmrstarlib.csviewer.**CSViewer**(*from_path*, *amino_acids=None*, *atoms=None*, *filename=None*, *csview_format='svg'*, *nmrstar_version='3'*)
Chemical Shifts Viewer uses chem_shifts_by_residue() method to get chemical shifts organized by residue and visualizes chemical shifts values using the Graphviz (http://www.graphviz.org/) DOT Languge description.

__init__(*from_path*, *amino_acids=None*, *atoms=None*, *filename=None*, *csview_format='svg'*, *nmrstar_version='3'*)
CSViewer initializer.

Parameters

- **from_path** (*str*) – Path to single NMR-STAR file or BMRB id.
- **amino_acids** (list or tuple) – Sequence of amino acids three letter codes, e.g. 'ALA', 'GLY', 'SER', etc. Leave as *None* to include everything.

- **atoms** (`list` or `tuple`) – Sequence of atom types, e.g. 'CA', 'CB', 'HA', etc. Leave as *None* to include everything.
- **filename** (*str*) – Output filename chemical shifts graph to be saved.
- **csview_format** (*str*) – *svg*, *png*, *pdf*. See http://www.graphviz.org/doc/info/output.html for all available formats.
- **nmrstar_version** (*str*) – Version of NMR-STAR format to use for look up chemichal shifts loop.

Returns None

Return type `None`

csview(*view=False*)

View chemical shift values organized by amino acid residue.

Parameters view (`True` or `False`) – Open in default image viewer or save file in current working directory quietly.

Returns None

Return type `None`

__weakref__

list of weak references to the object (if defined)

2.4 License

The MIT License (MIT)

Copyright (c) 2011 Morgan Astra, Hunter N.B. Moseley

Copyright (c) 2016 Andrey Smelter, Morgan Astra, Hunter N.B. Moseley

Permission is hereby granted, free of charge, to any person obtaining a copy of this software and associated documentation files (the "Software"), to deal in the Software without restriction, including without limitation the rights to use, copy, modify, merge, publish, distribute, sublicense, and/or sell copies of the Software, and to permit persons to whom the Software is furnished to do so, subject to the following conditions:

The above copyright notice and this permission notice shall be included in all copies or substantial portions of the Software.

THE SOFTWARE IS PROVIDED "AS IS", WITHOUT WARRANTY OF ANY KIND, EXPRESS OR IMPLIED, INCLUDING BUT NOT LIMITED TO THE WARRANTIES OF MERCHANTABILITY, FITNESS FOR A PARTICULAR PURPOSE AND NONINFRINGEMENT. IN NO EVENT SHALL THE AUTHORS OR COPYRIGHT HOLDERS BE LIABLE FOR ANY CLAIM, DAMAGES OR OTHER LIABILITY, WHETHER IN AN ACTION OF CONTRACT, TORT OR OTHERWISE, ARISING FROM, OUT OF OR IN CONNECTION WITH THE SOFTWARE OR THE USE OR OTHER DEALINGS IN THE SOFTWARE.

CHAPTER

THREE

INDICES AND TABLES

- *genindex*
- *modindex*
- *search*

PYTHON MODULE INDEX

n

nmrstarlib, 18
nmrstarlib.bmrblex, 22
nmrstarlib.converter, 23
nmrstarlib.csviewer, 27
nmrstarlib.nmrstarlib, 18

Symbols

__init__() (nmrstarlib.converter.Converter method), 26
__init__() (nmrstarlib.csviewer.CSViewer method), 27
__init__() (nmrstarlib.nmrstarlib.GenericFilePath method), 22
__init__() (nmrstarlib.nmrstarlib.StarFile method), 19
__weakref__ (nmrstarlib.converter.Converter attribute), 27
__weakref__ (nmrstarlib.csviewer.CSViewer attribute), 28
__weakref__ (nmrstarlib.nmrstarlib.GenericFilePath attribute), 22
_build_loop() (nmrstarlib.nmrstarlib.StarFile method), 19
_build_saveframe() (nmrstarlib.nmrstarlib.StarFile method), 19
_build_starfile() (nmrstarlib.nmrstarlib.StarFile method), 19
_generate_filenames() (in module nmrstarlib.nmrstarlib), 21
_generate_handles() (in module nmrstarlib.nmrstarlib), 22
_is_json() (nmrstarlib.nmrstarlib.StarFile static method), 21
_is_nmrstar() (nmrstarlib.nmrstarlib.StarFile static method), 21
_many_to_many() (nmrstarlib.converter.Converter method), 26
_one_to_one() (nmrstarlib.converter.Converter method), 26
_outputpath() (nmrstarlib.converter.Converter method), 27
_skip_saveframe() (nmrstarlib.nmrstarlib.StarFile method), 21
_to_bz2file() (nmrstarlib.converter.Converter method), 27
_to_dir() (nmrstarlib.converter.Converter method), 26
_to_gzipfile() (nmrstarlib.converter.Converter method), 27
_to_json() (nmrstarlib.nmrstarlib.StarFile method), 20
_to_nmrstar() (nmrstarlib.nmrstarlib.StarFile method), 21
_to_tarfile() (nmrstarlib.converter.Converter method), 27
_to_textfile() (nmrstarlib.converter.Converter method), 27
_to_zipfile() (nmrstarlib.converter.Converter method), 26

B

bmrblex() (in module nmrstarlib.bmrblex), 23

C

chem_shifts_by_residue() (nmrstarlib.nmrstarlib.StarFile method), 21
convert() (nmrstarlib.converter.Converter method), 26
Converter (class in nmrstarlib.converter), 26
csview() (nmrstarlib.csviewer.CSViewer method), 28
CSViewer (class in nmrstarlib.csviewer), 27

G

GenericFilePath (class in nmrstarlib.nmrstarlib), 22

I

is_compressed() (nmrstarlib.nmrstarlib.GenericFilePath static method), 22
is_url() (nmrstarlib.nmrstarlib.GenericFilePath static method), 22

N

nmrstarlib (module), 18
nmrstarlib.bmrblex (module), 22
nmrstarlib.converter (module), 23
nmrstarlib.csviewer (module), 27
nmrstarlib.nmrstarlib (module), 18

O

open() (nmrstarlib.nmrstarlib.GenericFilePath method), 22

P

print_loop() (nmrstarlib.nmrstarlib.StarFile method), 20
print_saveframe() (nmrstarlib.nmrstarlib.StarFile method), 20
print_starfile() (nmrstarlib.nmrstarlib.StarFile method), 20

R

read() (nmrstarlib.nmrstarlib.StarFile method), 19
read_files() (in module nmrstarlib.nmrstarlib), 22

S

StarFile (class in nmrstarlib.nmrstarlib), 19

U

update_constants() (in module nmrstarlib.nmrstarlib), 21

W

write() (nmrstarlib.nmrstarlib.StarFile method), 19
writestr() (nmrstarlib.nmrstarlib.StarFile method), 19
